# Role of Relaxation Time Scale in Noisy Signal Transduction

**DOI:** 10.1371/journal.pone.0123242

**Published:** 2015-05-08

**Authors:** Alok Kumar Maity, Pinaki Chaudhury, Suman K Banik

**Affiliations:** 1 Department of Chemistry, University of Calcutta, Kolkata, India; 2 Department of Chemistry, Bose Institute, Kolkata, India; Fondazione Edmund Mach, Research and Innovation Centre, ITALY

## Abstract

Intra-cellular fluctuations, mainly triggered by gene expression, are an inevitable phenomenon observed in living cells. It influences generation of phenotypic diversity in genetically identical cells. Such variation of cellular components is beneficial in some contexts but detrimental in others. To quantify the fluctuations in a gene product, we undertake an analytical scheme for studying few naturally abundant linear as well as branched chain network motifs. We solve the Langevin equations associated with each motif under the purview of linear noise approximation and derive the expressions for Fano factor and mutual information in close analytical form. Both quantifiable expressions exclusively depend on the relaxation time (decay rate constant) and steady state population of the network components. We investigate the effect of relaxation time constraints on Fano factor and mutual information to indentify a time scale domain where a network can recognize the fluctuations associated with the input signal more reliably. We also show how input population affects both quantities. We extend our calculation to long chain linear motif and show that with increasing chain length, the Fano factor value increases but the mutual information processing capability decreases. In this type of motif, the intermediate components act as a noise filter that tune up input fluctuations and maintain optimum fluctuations in the output. For branched chain motifs, both quantities vary within a large scale due to their network architecture and facilitate survival of living system in diverse environmental conditions.

## Introduction

Cell, the building block of every biological system, is capable of sensing extra-cellular as well as intra-cellular changes and responds accordingly using the mechanism of signal propagation through various network motifs [1–5]. The ubiquitous examples of biological motifs are signal transduction networks (STN) [6–10], gene transcription regulatory networks (GTRN) [11–15], metabolic reaction networks [16–18] and protein-protein interaction networks [19, 20]. The interactions between the different biochemical components of a motif are probabilistic in nature. Thus, fluctuations play an important role during the process of signal transduction [21–27]. The extent of performance of a network is measured by the response time, i.e., how fast the network output is changed with the fluctuating input stimuli [28]. If the network input-output relation follows a characteristic time scale then it could sense the changes (extra-cellular as well as intra-cellular) more precisely via components of a signal transduction motif. Consequently, some intra-cellular changes occur with the variation of input signal with few chemical modifications to optimize the effect of the input. One of the well studied STNs is mitogen activated protein kinase (MAPK) cascade, mostly observed in eukaryotic signaling pathway [6–8]. In MAPK cascade, external signal is processed through several steps via phosphorelay mechanism and fluctuations due to signaling molecules are subjected to modification at every step of the cascade. This phenomenon has been observed in various experimental and theoretical studies [29–32]. It has been shown that output fluctuations increase in an integrated way with the cascade length. On the other hand, the GTRN network is depicted by few nodes. These nodes represent regulatory genes and are connected via edges. A simple network can be constructed by considering two nodes representing two genes connected by a directed single edge. The edge signifies how the product of one gene (transcription factor) regulates the other gene and the direction of the edge represents the mode of regulation. In such case, transcription factor may act as the signaling molecule and plays a pivotal role in maintaining the transcription rate of a target gene by controlling the appropriate time scale and the amount of transcripts. GTRN motifs were initially identified in *E. coli* where few network motifs are much more common compared to the other random motifs [12, 33]. Later, these common motifs have been also observed in several other prokaryotes as well as in eukaryotes [2]. To understand the cellular physiology, it is thus essential to study the GTRN motifs at the single cell level. Cell shows phenotypic heterogeneity in genetically identical system due to fluctuations present within the cellular environment [15, 25, 34]. Fluctuations in the cellular component not only depend on the mean value of the component but also on the life time (relaxation time) [35]. Moreover, the relaxation rate of a biochemical component acts as a parameter which can be tuned during experiment [1]. In other words, tuning of relaxation time scale of each motif component provides a way to measure the amplification or suppression of fluctuations in each step of signal propagation. Recent development of monitoring protein degradation at the single cell level enables one to measure relaxation (degradation) time where fluctuations play an important role [36]. It is thus important to investigate how these networks perform under fluctuating condition. In the present paper we have studied few GTRN motifs to investigate the mode of functionality and their response in terms of the relaxation time scale. Our theoretical formalism takes care of the intrinsic noise associated with each biochemical reactions.

**Fig 1:**
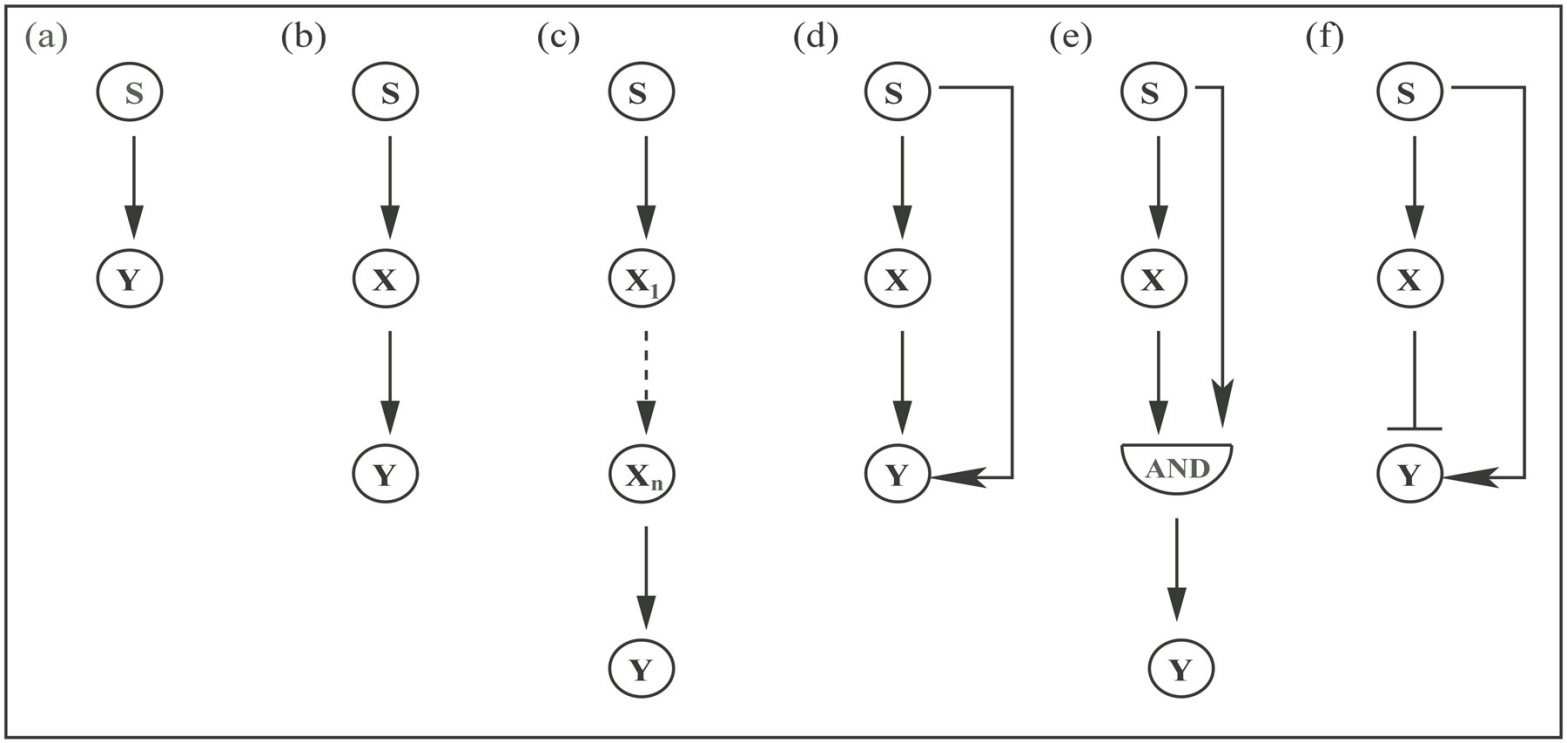
Schematic presentation of different GTRN motifs. (a) one step cascade (OSC), (b) two step cascade (TSC), (c) multi step cascade with *n* number of intermediate nodes, (d) OR coherent feed forward loop (OCFFL), (e) AND coherent feed forward loop (ACFFL) and (f) incoherent feed forward loop (ICFFL).

In the present communication, we focus on few naturally abundant GTRN network motifs. At first, we consider a simple linear one step cascade (OSC) and study the steady state dynamical behavior under stochastic framework. Two nodes and one edge are used to draw this motif, where each node represents a gene of a simple regulatory system (see Fig 1a). The edge indicates that one gene regulates the other via interaction of transcription factor with the promoter site of the target gene. Due to the direct interaction of the two nodes, this motif can be considered as a direct pathway for gene regulation or signal transmission. The next motif we undertake is a linear two step cascade (TSC) obtained from the previous motif by inserting a new node (gene) in between the two nodes of the one step network (see Fig 1b). In this case, the target gene is indirectly regulated by the input (transcription factor) that acts as a signal. Recent theoretical formalism reveals enhancement of signal processing capacity (fidelity) in TSC due to increase in the “biochemical noise” [37]. Furthermore, it has been shown that the relaxation time scale plays an important role in determining the fidelity.

Using both OSC and TSC network motifs, we then construct some biologically important motifs that belong to the group feed forward loop (FFL) (see Fig 1d–1f)). We compose these motifs by lateral combination of two linear cascades (OSC and TSC). In FFL, a transcription factor regulates the target gene directly (via OSC) as well as indirectly (via TSC) [1, 2, 12, 33, 38]. In these motifs, two transcription factors are present and each of these can show either positive (activation) or negative (repression) effect on the target gene. Therefore, eight different types of FFL are possible considering both effects. Among all the possible FFL, four of these are of coherent type and the remaining four are of incoherent type. The classification is done according to the overall sign of the regulatory motif, positive and negative sign for coherent and incoherent type, respectively. Experimentally, it has been shown that type-1 FFL has both coherent and incoherent nature and are ubiquitous. Due to this reason, we consider these two motifs in the present work. Type-1 coherent FFL has two sub-types depending upon the function of direct and indirect regulatory pathways on the promoter region of the target gene. When both transcription factors are required to express the target gene, the FFL motif behaves as an AND like gate (see Fig 1e). On the other hand, when one of the two transcription factors are sufficient to regulate the target gene, the FFL motif behaves as an OR like gate (see Fig 1d). At this point, it is important to mention that few theoretical studies under stochastic framework have been undertaken to understand the FFL motif [23, 39–41].

We use a Gaussian model (see Methods) to study the origin and consequence of stochasticity for all motifs considered in the present work. In all the motifs, fluctuations are carried forward from one node to the next one when signal is transduced along the direction of each edge. Thus, our main purpose here is the quantification of fluctuations in output signal for all motifs. Using an approximation technique (linear noise approximation [42–48]), we solve all dynamical equations and calculate the Fano factor (variance/mean) [49] expression by which we measure output fluctuations of each motif. Keeping this in mind we study the effect of relaxation time scale, i.e., lifetime of a network component on output fluctuations as it can provide knowledge about fluctuations propagation at each and every step of a cascade. We derive a time scale condition in which fluctuations in the input signal are filtered out by the intermediate component. Similarly, conditions have been figured out when fluctuations are enhanced. We also examine the effect of copy numbers of input signal on output fluctuations and show that it plays a vital role under some specific conditions. As all cascades process information of the input signal, we investigate the reliability of information flow through each cascade by measuring the mutual information between the input signal and the output [37, 50–53]. We calculate similar properties for FFL and identify biological significance of two sub types of coherent motifs.

## Methods

To start with, we consider few network motifs that represent GTRN. All the motifs that are taken into account in the present work are shown in Fig 1 where each circle represents a node and a straight line with an arrowhead connecting two different nodes represents an edge. The direction of an arrowhead denotes flow of signal from one node to the next one. The simplest linear signal transduction motif is modeled by two nodes, where S component acts as an input signal (transcription factor) which regulates the expression of a target gene Y (Fig 1a). The length of linear motif is increased further by incorporating another node X in between the S and the Y nodes. In such case, the S component regulates or transduces input signal to the Y component via the intermediate X (Fig 1b) [4, 54, 55]. We also consider a long chain linear motif (Fig 1c) by integrating *n* numbers of intermediate nodes within the simplest motif shown in Fig 1a.

Next, we focus on few branched chain network motifs that are constructed by lateral combination of the first two signal transduction motifs (one step and two step, Fig 1a and 1b, in different ways and are characterized as feed forward loop (FFL). Fig 1d represents coherent feed-forward loop of OR like gate (OCFFL), where the target gene Y is positively regulated by either the S or by the X component, both acting as transcription factors. On the other hand, for coherent feed forward loop of AND like gate (ACFFL), both S and X are essential to regulate the target gene Y positively (Fig 1e). In Fig 1f, the transcription factor S positively regulates the production of gene Y via direct pathway but represses the gene regulation via the X mediated indirect pathway and this motif is known as incoherent feed forward loop (ICFFL) [2, 33, 55, 56].

All the biochemical network motifs considered in Fig 1 consist of an input signal S and a output signal Y with an intermediate X except the long chain linear motif with *n* numbers of intermediate components (see Fig 1c). We describe the time dependent dynamics of the three chemical components by a set of generic coupled Langevin equations [42, 43, 47, 51] which may be of linear or non linear type depending on the kinetic schemes of a network motif considered in the present work

$$\begin{array}{ccc} \frac{ds}{dt} & = & f_{s}\left(s\right)-\tau_{s}^{-1}s+\xi_{s}\left(t\right), \end{array}$$ (1)

$$\begin{array}{ccc} \frac{dx}{dt} & = & f_{x}\left(s,x\right)-\tau_{x}^{-1}x+\xi_{x}\left(t\right), \end{array}$$ (2)

$$\begin{array}{ccc} \frac{dy}{dt} & = & f_{y}\left(s,x,y\right)-\tau_{y}^{-1}y+\xi_{y}\left(t\right). \end{array}$$ (3)

The first and the second terms on the right hand side of Eq (1) take care of synthesis and degradation of S, respectively, whereas *ξ*_*s*_(*t*) is the fluctuations associated with the input signal. In Eq (2), we consider the dynamics of intermediate X where *f*_*x*_(*s*, *x*) is the S and/or X mediated synthesis of X and $\tau_{x}^{-1}x$ takes care of degradation of X. As in Eq (1), *ξ*_*x*_(*t*) is the fluctuations associated with X. Similarly, the first and the second terms on the right hand side of Eq (3) presents S and/or X and/or Y mediated synthesis of output Y and degradation of the same, respectively, and *ξ*_*y*_(*t*) is the fluctuations associated with Y. Thus without loosing generality *f*_*i*_ and $\tau_{i}^{-1}$, in Eqs (1–3), represent the functional form of synthesis and degradation (inverse of life time *τ*_*i*_) rate constants of the *i*-th (*i* = *s*, *x*, *y*) chemical component, respectively. Here, *s*, *x* and *y* stand for the copy number of the chemical species S, X and Y, respectively, expressed in molecules/*V* where *V* is the unit cellular volume. This convention has been adopted for all the network motifs considered in the present work. The noise terms *ξ*_*i*_ are considered to be Gaussian white noise with zero mean, ⟨*ξ*_*i*_⟩ = 0. The noise strength or the variance associated with each noise term can be written as ⟨∣*ξ*_*i*_∣^2^⟩, quantified by the sum of production and decay rate [32, 43, 47, 57]. To be explicit, ⟨*ξ*_*i*_(*t*)*ξ*_*j*_(*t*′)⟩ = ⟨∣*ξ*_*i*_∣^2^⟩*δ*_*ij*_ *δ*(*t* − *t*′), where $\left\langle |\xi_{i}{|}^{2}\rangle =\langle f_{i}\rangle +\tau_{i}^{-1}\langle i\rangle =2\tau_{i}^{-1}\langle i\right\rangle$. In general, the variance associated with each noise term is a time dependent quantity [58, 59]. However, when the calculation is carried out at steady state one can use time independent expression of variance [32, 42, 43, 47] as considered in the present work. The cross-correlation between two noise terms is zero as the two kinetics are uncorrelated with each other. Considering that the copy number of each component is large at steady state, we study the dynamics of each motif (shown in Fig 1) at steady state within the purview of linear noise approximation [42–48]. Recent study has shown that linear noise approximation is valid for first order reaction kinetics as well as for bi-molecular reaction kinetics with large copy numbers [48]. It has been further shown to be applicable for chemical Langevin equation [59]. The dynamics of the signal transduction motifs shown in Fig 1 takes care of first order and/or bi-molecular reaction kinetics with high copy numbers, and has been theoretically formulated using coupled Langevin equations. Since the relaxation time of each component is small compared to the coarse grained (steady state) time scale, the Langevin equations we have adopted could satisfactorily explain the dynamics of each motif.

To solve the set of Langevin equations in a generalized way, we write these equations in the matrix form. To this end, we introduce two vectors *z* and *ξ* where *z* = (*s*, *x*, *y*) and *ξ* = (*ξ*_*s*_, *ξ*_*x*_, *ξ*_*y*_). Linearizing the Langevin equations around steady state and considering the change in the copy number due to fluctuations of each species from steady state to be very small, one can write *δz*(*t*) = *z*(*t*) − ⟨*z*⟩, where ⟨*z*⟩ is the steady state value of *z*. The linearized Langevin equation thus takes the form

$$\begin{array}{c} \frac{d\delta z}{dt}=J_{z=\langle z\rangle }\delta z\left(t\right)+\xi \left(t\right). \end{array}$$ (4)

Here, *J* is the Jacobian matrix evaluated at steady state. The diagonal elements of matrix *J* define the relaxation time of each component ($J_{ii}=-\tau_{i}^{-1}$) and the off-diagonal terms take care of interaction between the two components [45, 46, 57, 60]. Performing Fourier transformation of the linearized equation, we obtain

$$\begin{array}{c} i\omega \delta \tilde{z}\left(\omega \right)=J_{z=\langle z\rangle }\delta \tilde{z}\left(\omega \right)+\tilde{\xi }\left(\omega \right), \end{array}$$ (5)

where $\delta \tilde{z}(\omega )$ and $\tilde{\xi }(\omega )$ are Fourier transforms of *δz*(*t*) and *ξ*(*t*), respectively. The power spectra of the network components can be derived using Eq (5) [45–47, 61]

$$\begin{array}{c} 𝒮\left(\omega \right)={\left[i\omega I-J\right]}^{-1}H{\left[-i\omega I-J^{T}\right]}^{-1}, \end{array}$$ (6)

where *I* is the identity matrix and *J* ^*T*^ is the transpose of matrix *J*. The elements of matrix 𝓢 and matrix *H* are,

$$\begin{array}{ccc} {𝒮}_{ij}\left(\omega \right) & = & \langle \delta {\tilde{z}}_{i}\left(\omega \right)\delta {\tilde{z}}_{j}\left(-\omega \right)\rangle ,\\ H_{ij} & = & \left\langle {\tilde{\xi }}_{i}\left(\omega \right){\tilde{\xi }}_{j}\left(-\omega \right)\right\rangle =\left\langle \right|\xi_{i}{|}^{2}\rangle \delta_{ij}. \end{array}$$

The elements of matrix *H* thus stand for noise strength. Before proceeding further it is important to mention that to calculate the variance of linearized Langevin Eq (4) one may also use the Lyapunov matrix equation *JC* + *CJ* ^*T*^ + *H* = 0 where *C* is the covariance matrix [42, 45, 46]. Next, we perform the inverse Fourier transformation of the power spectra for every network component at steady state and evaluate the variance as well as covariance of the individual component and between two components, respectively. From the variance of output component, one can quantify the extent of fluctuations that are transduced by the final transcript of all the network motifs considered and the quantity of fluctuations can be defined in terms of Fano factor $\sigma_{y}^{2}/\langle y\rangle$, ratio of variance and population of Y component [49].

At this point it is important to mention that all the network motifs we have considered in the present work are regulated by a common transcription factor S and the output of each motif is Y. This led us to calculate the association in between the fluctuating input S and the output Y in terms of mutual information 𝓘(*s*, *y*) using Shannon’s formalism to check the reliability of all the network motifs [50, 53, 62, 63]

$$\begin{array}{c} ℐ\left(s,y\right)=\sum_{S}\sum_{Y}p\left(s,y\right)\log_{2}\frac{p(s,y)}{p(s)p(y)}. \end{array}$$ (7)

Here *p*(*s*, *y*) is the joint probability distribution function of the input S and the output Y. *p*(*s*) and *p*(*y*) are the marginal probability distribution functions of input S and output Y, respectively. In the present study, statistical properties of all the network motifs are evaluated at steady state where the fluctuations are considered to be Gaussian in nature. Under this constrain all motifs attain maximum possible entropy [50]. Since, for a Gaussian distribution function entropy depends only on the variance (*σ* ^2^) of the distribution, Eq (7) can be simplified in terms of the variance associated with the input S and the output Y and also the covariance between them. Moreover, in the present study we have adopted linear noise approximation that leads to Gaussian distribution. Thus for a jointly Gaussian distribution Eq (7) simplifies into [50, 53, 62, 63]

$$\begin{array}{c} ℐ\left(s,y\right)=\frac{1}{2}\log_{2}[1+\frac{\sigma_{sy}^{4}}{\sigma_{s}^{2}\sigma_{y}^{2}-\sigma_{sy}^{4}}], \end{array}$$ (8)

where $\sigma_{s}^{2}$ and $\sigma_{y}^{2}$ are the variance associated with the S and the Y component, respectively, and the covariance between them is given by $\sigma_{sy}^{2}$. Note that, theoretical calculation provides $\sigma_{s}^{2}=\langle s\rangle$, a signature of simple birth-death process. In the present work evaluation of mutual information is based on Gaussian nature of the noise processes. A more general theoretical formalism has been proposed by Bowsher et al [37] which is independent of the nature of distribution of the input signal. The quantity $\sigma_{sy}^{4}/(\sigma_{s}^{2}\sigma_{y}^{2}-\sigma_{sy}^{4})$ in Eq (8) is known as signal-to-noise ratio in the literature and is related to fidelity of the signal transduction mechanism [37, 52].

To check the validity of our theoretical analysis, we perform numerical calculation using stochastic simulation algorithm [64, 65]. For reaction schemes, propensities and rate constants associated with each motif we refer to S1 Table. While performing simulation we have expressed the components (*s*, *x*, and *y*) in molecules/*V*. In addition, the rate constants have been expressed in min^−1^. During numerical evaluation of mutual information we have calculated two variances ($\sigma_{s}^{2}$ and $\sigma_{y}^{2}$) and one covariance ($\sigma_{sy}^{2}$) using the values *s* and *y* at steady state. These values are then used in Eq (8) to calculate mutual information. Furthermore, we also investigate the validity of using approximate Gaussian expression (Eq (8)) by numerical calculation of Eq (7). To this end, data generated by stochastic simulation algorithm [64, 65] allows us to generate the distribution functions *p*(*s*), *p*(*y*) and *p*(*s*, *y*) by employing the binning technique with a smallest bin size of 1. In the calculation of mutual information using theoretical and experimental data, specially the last one, bias estimation has a significant role [52, 66]. It depends on both bin size and number of sample size (number of trajectories generated from stochastic simulation). A biased mutual information converges to unbiased one for smallest bin size as well as for large number of sample size. In the present work we have used 10^5^ trajectories and bin size of 1 while calculating the mutual information. Due to this reason we do not employ any bias correction explicitly. To carry out the stochastic analysis we have used a set of biologically relevant parameters. Most of the parameters have been considered in the present work while keeping in mind the dynamics of the network motifs in prokaryotic system [1, 47, 55, 67].

## Results and Discussion

In the following subsections, we execute individual study of each network motif as well as perform a comparative study of all the motifs. From the Fano factor expression, one can discriminate the origin of fluctuations in a motif. We identify the network that faces maximum fluctuating environment under a definite condition and characterize the favorable circumstances in which it can transduce the information of the input signal more reliably.

### One step cascade

As one step cascade (OSC) is the simplest unit of addressing signal transduction motifs, we initially start with this simple motif. In this motif, the input signal S directly regulates the target gene Y. For the sake of simplicity, we consider that S is constitutively active and linearly regulates the target gene leading to the formation of Y. The stochastic kinetics in Langevin equation formalism is given by

$$\begin{array}{ccc} \frac{ds}{dt} & = & k_{1}-\tau_{s}^{-1}s+\xi_{s}\left(t\right), \end{array}$$ (9)

$$\begin{array}{ccc} \frac{dy}{dt} & = & k_{3}s-\tau_{y}^{-1}y+\xi_{y}\left(t\right), \end{array}$$ (10)

where *k*_1_ and *k*_3_ are the synthesis rate for S and Y, respectively. The degradation rates for the same components are given by $\tau_{s}^{-1}$ and $\tau_{y}^{-1}$, respectively. *ξ*_*s*_(*t*) and *ξ*_*y*_(*t*) are Gaussian white noise terms with zero mean ⟨*ξ*_*s*_(*t*)⟩ = ⟨*ξ*_*y*_(*t*)⟩ = 0. The respective noise strengths are given by $\left\langle \xi_{s}(t)\xi_{s}(t\prime )\rangle =2\tau_{s}^{-1}\langle s\rangle \delta (t-t\prime \right)$ and $\left\langle \xi_{y}(t)\xi_{y}(t\prime )\rangle =2\tau_{y}^{-1}\langle y\rangle \delta (t-t\prime \right)$, respectively. In addition, both noise processes are uncorrelated, ⟨*ξ*_*s*_(*t*)*ξ*_*y*_(*t*′)⟩ = ⟨*ξ*_*y*_(*t*)*ξ*_*s*_(*t*′)⟩ = 0. We solve Eqs (9–10) using analytical scheme described in Methods section to calculate the variance associated with the output Y and co-variance between the input signal S and the output Y [42–45, 47, 48]

$$\begin{array}{c} \sigma_{y}^{2}=\left\langle y\right\rangle +\frac{\tau_{y}^{-1}{\left\langle y\right\rangle }^{2}}{\left(\tau_{s}^{-1}+\tau_{y}^{-1}\right)\left\langle s\right\rangle },\sigma_{sy}^{2}=\frac{\tau_{y}^{-1}\left\langle y\right\rangle }{\tau_{s}^{-1}+\tau_{y}^{-1}}. \end{array}$$ (11)

In the above expression of variance $\sigma_{y}^{2}$, the first part on the right hand side arises due to intrinsic fluctuations in Y and the second part is responsible for extrinsic fluctuations, incorporated into the output Y through the input S during gene regulation. The total variance in this motif is expressed in terms of output variance $\sigma_{y}^{2}$ that follows spectral addition rule, sum of external and internal fluctuations [32, 57, 68–71]. At this point, it is important to mention that two fluctuating terms originating from different sources have been also calculated for the oscillatory system [72]. In this study, we are interested in Fano factor as well as in information propagation through the cascade with the variation of system’s relaxation times as well as steady state population ⟨*s*⟩ of the input component S, as both $\sigma_{y}^{2}$ and $\sigma_{sy}^{2}$ depend only on the time scale when the steady state value of both components ⟨*s*⟩ and ⟨*y*⟩ are kept fixed followed by a constant $k_{1}/\tau_{s}^{-1}$ and $k_{3}/\tau_{y}^{-1}$ ratio. Similarly, for constant relaxation times, both expressions vary with the steady state populations ⟨*s*⟩ and ⟨*y*⟩. In this motif, we have two relaxation time scales *τ*_*s*_ (input) and *τ*_*y*_ (output). These two time scales lead to three possible limiting conditions for which we get three different modified expressions for Fano factor ($\sigma_{y}^{2}/\langle y\rangle$) as well as for co-variance (see Table 1).

**Table 1:** Modified form of the analytical expression given by Eq (11) for OSC motif.

	*τ*_*s*_ ≫ *τ*_*y*_	*τ*_*s*_ ≈ *τ*_*y*_	*τ*_*s*_ ≪ *τ*_*y*_
Fano factor	$1+\frac{\langle y\rangle }{\langle s\rangle }$	$1+0.5\frac{\langle y\rangle }{\langle s\rangle }$	$1+\frac{\tau_{s}\langle y\rangle }{\tau_{y}\langle s\rangle }$
$\sigma_{sy}^{2}$	〈*y*〉	0.5〈*y*〉	$\frac{\tau_{s}}{\tau_{y}}\langle y\rangle$

Fano factor ($\sigma_{y}^{2}/\langle y\rangle$) and co-variance ($\sigma_{sy}^{2}$) for different relaxation time limit are shown in this table.

In Table 1, Fano factor and $\sigma_{sy}^{2}$ values are maximum at the time limit *τ*_*s*_ ≫ *τ*_*y*_ whereas, both are minimum at the time limit *τ*_*s*_ ≪ *τ*_*y*_. These results reveal that the effect of input fluctuations into the output fluctuations gets maximized if the input signal relaxes at a much slower rate compared to the output signal ($\tau_{s}^{-1}\ll \tau_{y}^{-1}$) and will be minimized for faster input relaxation rate compared to the output one ($\tau_{s}^{-1}\gg \tau_{y}^{-1}$). However, the output signal faces an intermediate level of fluctuations when both signals have comparable relaxation rate ($\tau_{s}^{-1}\approx \tau_{y}^{-1}$).

**Fig 2:**
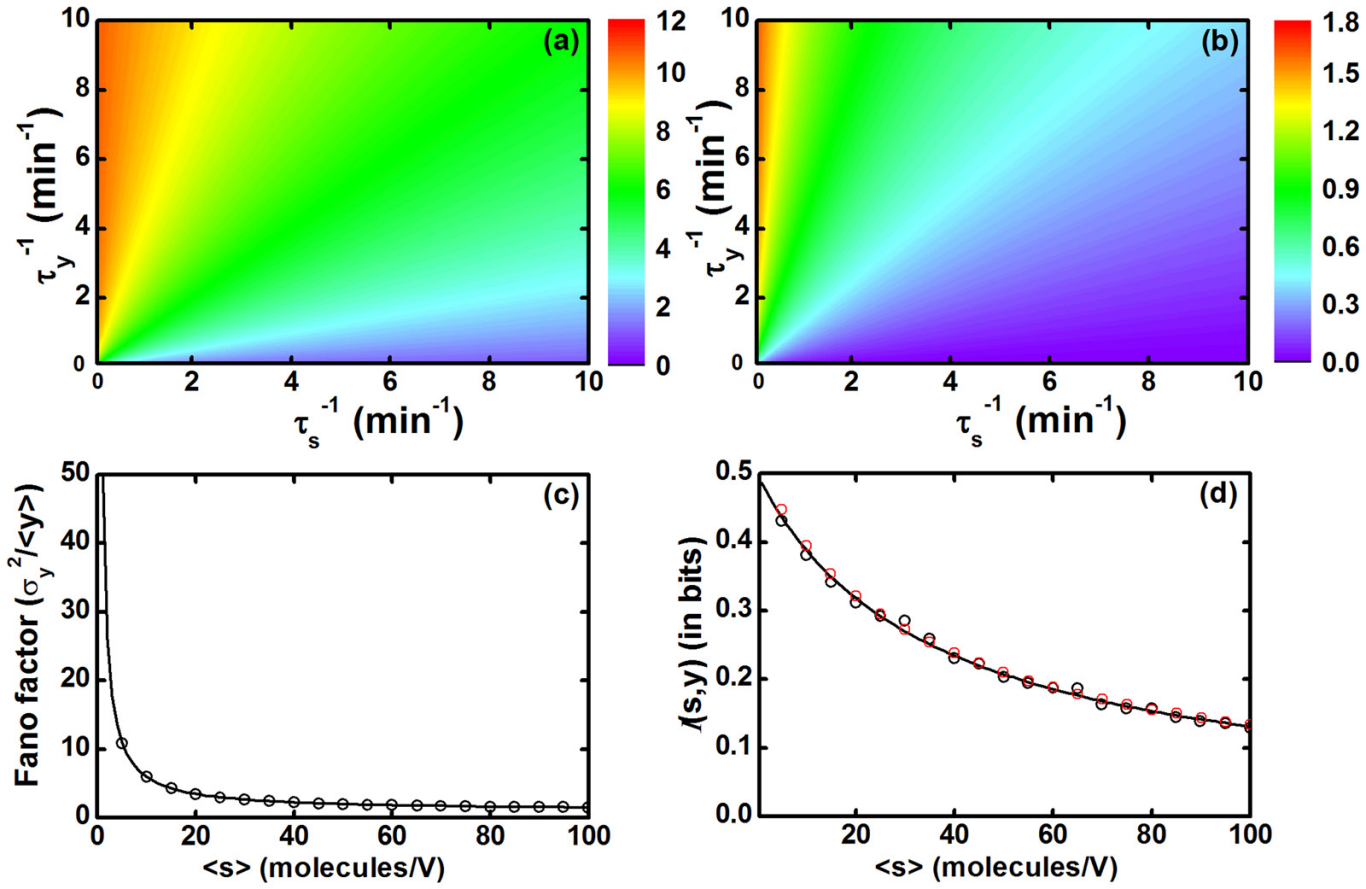
The OSC. (a, b) Two dimensional maps of Fano factor and mutual information 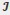
(*s*, *y*), respectively, as a function of two relaxation rate constants $\tau_{y}^{-1}$ and $\tau_{s}^{-1}$ for the ratio $k_{1}/\tau_{s}^{-1}=k_{3}/\tau_{y}^{-1}=10$. (c) Fano factor and (d) mutual information 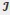
(*s*, *y*) profiles as a function of mean input signal level ⟨*s*⟩ expressed in molecules/unit cellular volume (*V*). Parameters used are $\tau_{s}^{-1}=\tau_{y}^{-1}=1.0$ min^−1^ and *k*_3_ = 100/*k*_1_ min^−1^. The symbols are generated using stochastic simulation algorithm [64, 65] and the lines are due to theoretical calculation. In the plot of mutual information the red and black symbols are due to Eq (7) and Eq (8), respectively.

For faster fluctuations in the input component (*τ*_*s*_ ≪ *τ*_*y*_), the target gene cannot sense the rapid concentration changes of the input signal and shows an average response. In such a case, external fluctuations have no significant contribution in the fluctuations of output and consequently, suppression of the output fluctuations is executed and minimum Fano factor value is obtained. In this connection, it is important to mention that for very large *τ*_*y*_, the ratio *τ*_*s*_/*τ*_*y*_ is very low (≪ 1) and contribution of extrinsic fluctuations becomes insignificant in the total output fluctuations. Therefore, output fluctuations only depend on the mean steady state value of the target gene and the network motif follows a Poisson statistic, i.e., behaves like a simple birth-death process (Fano factor $\sigma_{y}^{2}/\langle y\rangle =1$). However, the target gene successfully characterizes the concentration change of signaling molecule for slower input fluctuations (*τ*_*s*_ ≫ *τ*_*y*_). In this time scale, the OSC motif transduces extracellular or upstream signal reliably and provides an exact response with the achievement of maximum Fano factor. When both time scales are approximately equal (*τ*_*s*_ ≈ *τ*_*y*_), extrinsic fluctuations get partially incorporated into the total fluctuations and give an intermediate Fano factor value which is in between the two extreme cases, slowest and fastest input fluctuations. It seems apparent that the motif can sense external fluctuations with a greater extent in the nearly equal relaxation time scales but our result does not show that. As both reactions are stochastic in nature, they have probabilistic character that executes two chance factors. In one situation, the target gene can properly characterize the input fluctuations and in the other situation, it fails to characterize the same. This results into a statistical weightage value of 0.5 in the contribution of extrinsic fluctuations in the Fano factor expression. A similar kind of time scale effect is also shown by the co-variance expression which governs the mutual information transduction. In Fig 2a and 2b, we show surface plots of Fano factor and mutual information, respectively, as a function of two relaxation rate constants, $\tau_{y}^{-1}$ and $\tau_{s}^{-1}$, where we maintain the steady state population of both components by a constant parameters ratio $k_{1}/\tau_{s}^{-1}=k_{3}/\tau_{3}^{-1}=10$. Fig 2a shows that the maximum Fano factor value is attained by the motif only at very low input relaxation rate constant compared to the output one. Along diagonal axis both rate constants are approximately equal. Hence, the magnitude of Fano factor is within an intermediate range. The minimum level of Fano factor value is observed at very high input rate constant compared to the output one. In Fig 2b, the 2d-plot of mutual information also varies in a similar fashion as the Fano factor plot. As the OSC motif performs under the definite input fluctuations, the information transduction capability of the motif is mainly characterized on the basis of input-output relaxation time scales. As a result, the motif can transduce the input information more reliably at faster relaxation time scale of the output component among all the relaxation time scales of output component (see the three limiting conditions in Table 1).

For the three relaxation time limiting cases given in Table 1, the Fano factor expression also depends on the steady state population of the network components. However, from the Fano factor expression it is clear that extrinsic fluctuations can contribute an appreciable amount in the total output noise if the steady state population of output component is much higher than the input one. This means that for a highly populated input signal, the fluctuations in the input do not have significant effect on the regulation of the target gene. Consequently, the regulated gene works under an apparently constant level of the input signal. Therefore, the motif shows low level of Fano factor value even if it belongs to the relaxation time scale limit where maximum input fluctuations are transduced. In Fig 2c, we show the Fano factor associated with the variation of population of the signaling molecule S. In this plot, a sharp exponential decay of the Fano factor is observed as the steady state population of the input signal increases. In Fig 2d, mutual information 𝓘(*s*, *y*) is plotted as a function of the input signal. In Fig 2c and 2d, population level of the input signal is systematically increased by increasing the synthesis rate constant *k*_1_ whereas, the steady state population of the output Y is kept fixed by simultaneous change of the synthesis rate constant *k*_3_, followed by a mathematical relation *k*_3_ = 100/*k*_1_ min^−1^. The rest of the parameter values used are $\tau_{1}^{-1}=\tau_{3}^{-1}=1.0$ min^−1^. In the mutual information plot (Fig 2d), sharp exponential decay is not observed as in Fig 2c). This happens due to the absence of explicit dependence of signaling molecule in the expression of co-variance (see Eq (10)) and for keeping ⟨*y*⟩ fixed. In addition, in the expression of mutual information (see Eq (8)), the input signal has a predominant effect due to $\sigma_{s}^{2}$ that overcomes the decreasing effect of $\sigma_{y}^{2}$ associated with Y.

Based on the analysis provided in the aforesaid discussion, one can compare the OSC motif with the well known motif of gene transcription and translation machinery. Here, mRNA, the product of transcription can be considered as signaling component S generated from a fully induced or a constitutive promoter at a constant rate. Similarly, protein, the product of translation plays the role of component Y translated from mRNA. If one does not take into account the genetic switching steps (on/off state of a promoter) then one can easily compare the gene regulation network with the OSC motif where mRNA (S) and protein (Y) represent the input and the output component, respectively. From the conditions presented in Table 1, one can conclude that the gene regulation motif can only attain a low level of fluctuations in the protein level through high population and relaxation rate constant of the input component compared to the output one. Thus, fluctuations in the protein Y, the gene product, are modulated via kinetic parameter as well as deterministic population of mRNA which acts as signaling component S. Protein molecule shows minimum fluctuations under the constraints of large number of mRNA with very low average lifetime *τ*_*s*_. These phenomena have been verified extensively via experimental and theoretical studies [21, 67, 68, 70, 73–78]. Similarly, an extensive study on noise propagation in eukaryotic gene expression has been accomplished using data from two high-throughput experiments where Fraser et al [75] have observed that the production of essential and complex-forming proteins implicate low level of fluctuations compared to other proteins and this low level of fluctuations is attained via high transcription rate and low translation efficiency. Due to a high transcription rate, a large amount of mRNA is generated. While doing the analysis, they have used the definition of the translation efficiency, ratio of protein synthesis and mRNA decay rate constant. Translation efficiency can be minimized for very high decay (relaxation) rate constant of mRNA molecule, i.e., very short lifetime under a constant protein production rate. From our calculation, we also get an equivalent result that successfully explicates their noble findings.

### Two step cascade

In two step cascade (TSC) motif (see Fig 1b), the output component Y is indirectly regulated by the input component S via an intermediate component X. As the dynamical equation for the input signal S is same as in the previous motif (Eq (9)), we do not write it here explicitly. The linear Langevin equations for the remaining two components X and Y are given as

$$\begin{array}{ccc} \frac{dx}{dt} & = & k_{2}s-\tau_{x}^{-1}x+\xi_{x}\left(t\right), \end{array}$$ (12)

$$\begin{array}{ccc} \frac{dy}{dt} & = & k_{3}x-\tau_{y}^{-1}y+\xi_{y}\left(t\right). \end{array}$$ (13)

In the above equations, *k*_2_ and *k*_3_ are the synthesis rate constants of X and Y component, respectively. $\tau_{i}^{-1}$ and *ξ*_*i*_(*t*) (*i* = *x*, *y*) are decay rate constants and Langevin force terms of the respective component. As in the OSC, here the three noise terms (*ξ*_*s*_, *ξ*_*x*_ and *ξ*_*y*_) are of Gaussian white type with zero mean ⟨*ξ*_*s*_(*t*)⟩ = ⟨*ξ*_*x*_(*t*)⟩ = ⟨*ξ*_*y*_(*t*)⟩ = 0. The respective noise strengths are given by $\left\langle \xi_{s}(t)\xi_{s}(t\prime )\rangle =2\tau_{s}^{-1}\langle s\rangle \delta (t-t\prime \right)$, $\left\langle \xi_{x}(t)\xi_{x}(t\prime )\rangle =2\tau_{x}^{-1}\langle x\rangle \delta (t-t\prime \right)$ and $\left\langle \xi_{y}(t)\xi_{y}(t\prime )\rangle =2\tau_{y}^{-1}\langle y\rangle \delta (t-t\prime \right)$, respectively. In addition, the three noise processes are uncorrelated, ⟨*ξ*_*s*_(*t*)*ξ*_*x*_(*t*′)⟩ = ⟨*ξ*_*x*_(*t*)*ξ*_*s*_(*t*′)⟩ = 0, ⟨*ξ*_*x*_(*t*)*ξ*_*y*_(*t*′)⟩ = ⟨*ξ*_*y*_(*t*)*ξ*_*x*_(*t*′)⟩ = 0 and ⟨*ξ*_*s*_(*t*)*ξ*_*y*_(*t*′)⟩ = ⟨*ξ*_*y*_(*t*)*ξ*_*s*_(*t*′)⟩ = 0. Solving the Langevin equations in a similar manner [42–45, 47, 48], we arrive at the expression of variance and co-variance of the output component Y

$$\begin{array}{ccc} \sigma_{y}^{2} & = & \left\langle y\right\rangle +\frac{\tau_{y}^{-1}{\left\langle y\right\rangle }^{2}}{\left(\tau_{x}^{-1}+\tau_{y}^{-1}\right)\left\langle x\right\rangle }\\ & & +\frac{\tau_{x}^{-1}\tau_{y}^{-1}\left(\tau_{s}^{-1}+\tau_{x}^{-1}+\tau_{y}^{-1}\right){\left\langle y\right\rangle }^{2}}{\left(\tau_{s}^{-1}+\tau_{x}^{-1}\right)\left(\tau_{x}^{-1}+\tau_{y}^{-1}\right)\left(\tau_{s}^{-1}+\tau_{y}^{-1}\right)\left\langle s\right\rangle },\\ \sigma_{sy}^{2} & = & \frac{\tau_{x}^{-1}\tau_{y}^{-1}\left\langle y\right\rangle }{\left(\tau_{s}^{-1}+\tau_{x}^{-1}\right)\left(\tau_{s}^{-1}+\tau_{y}^{-1}\right)}. \end{array}$$ (14)

The first term on the right hand side of the variance $\sigma_{y}^{2}$ reveals the intrinsic fluctuations in the output component Y. The second and the third terms of the expression originate due to the fluctuations in the X and the S component, respectively. Compared to the OSC motif, an extra noise term appears in the variance which originates due to the addition of intermediate component X. Hence, the magnitude of total output fluctuations in TSC becomes higher compared to the OSC. If one inserts a new intermediate component into the TSC motif, the output fluctuations will increase further. This indicates that the output fluctuations are increased with the augmentation of cascade length. Such fluctuations integration character in each step of a cascade has been verified earlier both experimentally and theoretically [29–32]. In spite of these fluctuations enhancement property of long chain cascade networks, some long chain network motifs like MAPK signaling pathways as well as GTRNs are identified in living systems, where external signal gets transduced with great accuracy. This is an unusual but interesting aspect of living beings that promotes an extra curiosity to study signaling pathways to understand the execution of high precision signal transduction in highly fluctuating environment. While considering the dynamics of TSC, we try to decipher the criteria that leads to the understanding of the formation of final output Y under the condition of optimum fluctuations as per the system’s permissibility. In Eq (12), both expressions depend on the three relaxation rate constants $\tau_{s}^{-1}$, $\tau_{x}^{-1}$ and $\tau_{y}^{-1}$ as well as on the steady state population level of the network components (⟨*s*⟩, ⟨*x*⟩ and ⟨*y*⟩). Possible combinations of three relaxation times (*τ*_*s*_, *τ*_*x*_ and *τ*_*y*_) leads to nine different modified expressions of Fano factor ($\sigma_{y}^{2}/\langle y\rangle$) and co-variance ($\sigma_{sy}^{2}$), shown in Table 2.

**Table 2:** Modified forms of the analytical solution (Eq (14)) of TSC motif.

		*τ*_*x*_ ≫ *τ*_*y*_	*τ*_*x*_ ≈ *τ*_*y*_	*τ*_*x*_ ≪ *τ*_*y*_
*τ*_*s*_ ≫ *τ*_*x*_	Fano factor	$1+\frac{\left\langle y\right\rangle }{\left\langle x\right\rangle }+\frac{\left\langle y\right\rangle }{\left\langle s\right\rangle }$	$1+0.5\frac{\left\langle y\right\rangle }{\left\langle x\right\rangle }+\frac{\left\langle y\right\rangle }{\left\langle s\right\rangle }$	$1+\frac{\tau_{x}\langle y\rangle }{\tau_{y}\langle x\rangle }+\rho \frac{\left\langle y\right\rangle }{\left\langle s\right\rangle }$
	$\sigma_{sy}^{2}$	⟨*y*⟩	⟨*y*⟩	*ρ*⟨*y*⟩
*τ*_*s*_ ≈ *τ*_*x*_	Fano factor	$1+\frac{\left\langle y\right\rangle }{\left\langle x\right\rangle }+0.5\frac{\left\langle y\right\rangle }{\left\langle s\right\rangle }$	$1+0.5\frac{\left\langle y\right\rangle }{\left\langle x\right\rangle }+\frac{3\langle y\rangle }{8\langle s\rangle }$	$1+\frac{\tau_{x}\langle y\rangle }{\tau_{y}\langle x\rangle }+\frac{\tau_{x}\langle y\rangle }{\tau_{y}\langle s\rangle }$
	$\sigma_{sy}^{2}$	0.5⟨*y*⟩	0.25⟨*y*⟩	$0.5\frac{\tau_{s}\langle y\rangle }{\tau_{y}}$
*τ*_*s*_ ≪ *τ*_*x*_	Fano factor	$1+\frac{\left\langle y\right\rangle }{\left\langle x\right\rangle }+\frac{\tau_{s}\langle y\rangle }{\tau_{x}\langle s\rangle }$	$1+0.5\frac{\left\langle y\right\rangle }{\left\langle x\right\rangle }+0.5\frac{\tau_{s}\langle y\rangle }{\tau_{x}\langle s\rangle }$	$1+\frac{\tau_{x}\langle y\rangle }{\tau_{y}\langle x\rangle }+\frac{\tau_{s}\langle y\rangle }{\tau_{y}\langle s\rangle }$
	$\sigma_{sy}^{2}$	$\frac{\tau_{s}\rho \langle y\rangle }{\tau_{x}}$	$\frac{\tau_{s}^{2}\langle y\rangle }{\tau_{x}\tau_{y}}$	$\frac{\tau_{s}^{2}\langle y\rangle }{\tau_{x}\tau_{y}}$

Fano factor ($\sigma_{y}^{2}/\langle y\rangle$) and co-variance ($\sigma_{sy}^{2}$) at different relaxation time limits are shown with *ρ* = *τ*_*s*_/(*τ*_*s*_ + *τ*_*y*_) ⩽ 1.

**Fig 3:**
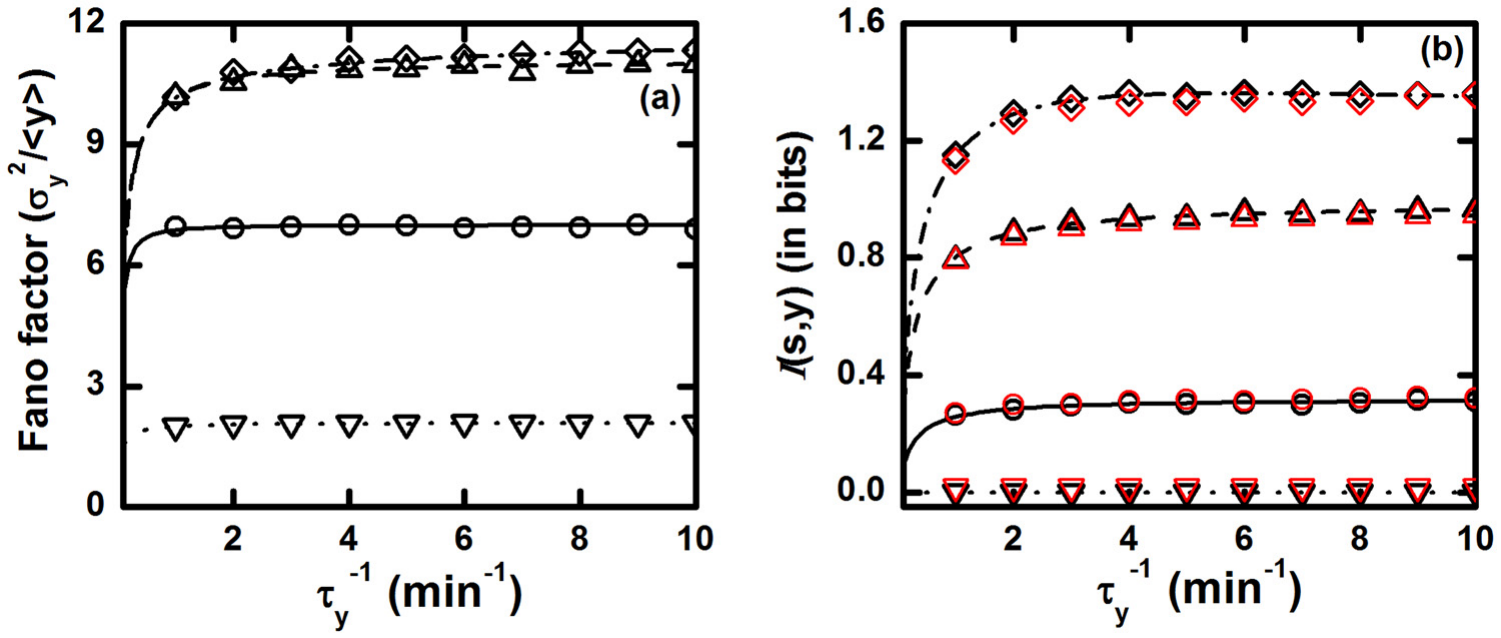
The TSC. (a) Fano factor and (b) mutual information 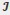
(*s*, *y*) profiles as a function of relaxation rate constant $\tau_{y}^{-1}$ of Y component. $k_{1}/\tau_{s}^{-1}=k_{2}/\tau_{x}^{-1}=10$ and $k_{3}/\tau_{y}^{-1}=1.0$ are maintained throughout these plots, so that steady state population of all the components remains unaltered. In both plots, for solid (with open circles), dashed (with open upward triangle), dotted (with open downward triangle) and dash dotted (with open diamond) lines we have used the relations $\tau_{s}^{-1}=\tau_{x}^{-1}=0.1$ min^−1^, $\tau_{s}^{-1}=\tau_{x}^{-1}/10=0.1$ min^−1^, $\tau_{s}^{-1}/100=\tau_{x}^{-1}=0.1$ min^−1^ and $\tau_{s}^{-1}=\tau_{x}^{-1}/100=0.1$ min^−1^, respectively. The symbols are generated using stochastic simulation algorithm [64, 65] and the lines are due to theoretical calculation. In the plot of mutual information the red and black symbols are due to Eq (7) and Eq (8), respectively.

From the modified expressions given in Table 2 it is easy to identify the maximum, intermediate and minimum value of Fano factor and co-variance under the condition *τ*_*s*_ ≫ *τ*_*x*_ ≫ *τ*_*y*_, *τ*_*s*_ ≈ *τ*_*x*_ ≈ *τ*_*y*_ and *τ*_*s*_ ≪ *τ*_*x*_ ≪ *τ*_*y*_, respectively. In all the expressions, effect of the population level of the input and the intermediate component on the output is clearly visible. As our main focus in the present study is to characterize the effect of relaxation time scales in terms of Fano factor and co-variance, we fix the steady state population of all the network components using the relations $k_{1}/\tau_{s}^{-1}=k_{2}/\tau_{x}^{-1}=10$ and $k_{3}/\tau_{y}^{-1}=1.0$. In Fig 3, we show Fano factor and mutual information for the TSC motif as a function of output relaxation rate constant $\tau_{y}^{-1}$ for different values of $\tau_{s}^{-1}$ and $\tau_{x}^{-1}$.

From the plot shown in Fig 3a, it is clear that Fano factor value increases with the increment of output relaxation rate constant $\tau_{y}^{-1}$ for all sets of parameter values. As the output component Y can track the comparably slower fluctuations of upstream input signal efficiently, fluctuations of the output component increases proportionately with its relaxation rate constant. The additive nature of fluctuations assists to amplify the Fano Factor value through total output as a function of $\tau_{y}^{-1}$ and the trend is persistent for all the four sets of parameter values used to draw Fig 3a. Among the four sets of parameters, higher Fano factor values are achieved by two sets of parameters. From the parameter sets $\tau_{s}^{-1}=\tau_{x}^{-1}/10=0.1$ min^−1^ and $\tau_{s}^{-1}=\tau_{x}^{-1}/100=0.1$ min^−1^, it is evident that the intermediate component X has faster fluctuations rate than the input component S ($\tau_{x}^{-1}\gg \tau_{s}^{-1}$). As a result, it becomes possible for X to characterize the fluctuations in S. At the equal relaxation time scale limits of S and X ($\tau_{s}^{-1}=\tau_{x}^{-1}=0.1$ min^−1^), we get an intermediate Fano factor profile and a minimum profile of the same is obtained for $\tau_{s}^{-1}/100=\tau_{x}^{-1}=0.1$ min^−1^. This happens as the S component fluctuates in a faster time scale compared to the X component which, in turn, forbids X to differentiate the concentration change in S. As a result, X is unable to carry forward the variation in signal (due to S) to the Y component of the TSC motif. In this relaxation time scale limit, the intermediate X acts as a low pass filter that passes only low-frequency input signal. For this reason, highly fluctuating (high-frequency) input signal is impeded by the intermediate component that inhibits fluctuations propagation through the motif.

In Fig 3b, mutual information 𝓘(*s*, *y*) flow varies with the relaxation rate of Y and we observe that TSC motif transduces information more reliably at faster fluctuations in Y due to proper characterization of the input signal variation and is applicable for different choice of parameters sets. For the four sets of parameters, mutual information follows the increasing trend of Fano factor but, it is important to mention that for $\tau_{s}^{-1}/100=\tau_{x}^{-1}=0.1$ min^−1^, 𝓘(*s*, *y*) value is almost zero. This happens due to slow fluctuations in X compared to S which impedes the flow of information of the input signal into the downstream component. As a result, the intermediate component cannot discriminate the variation of external signal and gives a constant response to the change in the environment. Although the Fano factor is minimum at this time frame, the TSC motif is unable to adapt due to the approximately zero mutual information processing ability. Hence, the network motif opts for a relaxation time scale where both fluctuations and mutual information processing capacity adopt an optimum value. Our observation of enhancement of information processing capacity, in a TSC motif, with increase in fluctuations is in agreement with recent theoretical formalism of Bowsher et al [37].

Next, we extend our calculation for a generalized motif of linear long chain cascade (see Fig 1c) considering *n* numbers of different intermediate components (X_1_, X_2_, .., X_*n*_) that are present in between the input and the output component. Using the concept of TSC motif, we obtain the expressions of Fano factor for three limiting relaxation rate conditions as

$$\begin{array}{ccc} & & 1+\frac{\left\langle y\right\rangle }{\left\langle x_{n}\right\rangle }+\cdots +\frac{\left\langle y\right\rangle }{\left\langle x_{1}\right\rangle }+\frac{\left\langle y\right\rangle }{\left\langle s\right\rangle }\\ & & \;\text{for}\;\tau_{y}⪢\tau_{x_{n}}⪢\cdots ⪢\tau_{x_{1}}⪢\tau_{s},\\ & & 1+c_{n}\frac{\left\langle y\right\rangle }{\left\langle x_{n}\right\rangle }+\cdots +c_{1}\frac{\left\langle y\right\rangle }{\left\langle x_{1}\right\rangle }+c_{s}\frac{\left\langle y\right\rangle }{\left\langle s\right\rangle }\\ & & \;\text{for}\;\tau_{y}\approx \tau_{x_{n}}\approx \cdots \approx \tau_{x_{1}}\approx \tau_{s},\\ & & 1+\frac{\tau_{x_{n}}\left\langle y\right\rangle }{\tau_{y}\left\langle x_{n}\right\rangle }+\cdots +\frac{\tau_{x_{1}}\left\langle y\right\rangle }{\tau_{y}\left\langle x_{1}\right\rangle }+\frac{\tau_{s}\left\langle y\right\rangle }{\tau_{y}\left\langle s\right\rangle }\\ & & \;\text{for}\;\tau_{y}⪡\tau_{x_{n}}⪡\cdots ⪡\tau_{x_{1}}⪡\tau_{s}. \end{array}$$

where *c*_1_, ⋯, *c*_*n*_ ⩽ 0.5, *c*_*s*_ ⩽ 0.5 and *τ*_*x*_1__, *τ*_*x*_2__, ⋯, *τ*_*x*_*n*__ are the relaxation time of the X_1_, X_2_, ⋯, X_*n*_ component, respectively. The expressions given above are three simplified forms of Fano factor expressions for linear long chain signal transduction motif or GTRN cascade with *n* number of different intermediate components. The main motivation to evaluate these simplified forms is that using the expressions, one can easily get a gross quantitative idea about the output fluctuations of any linear chain network cascade with large number of intermediate components. If one knows the steady state population level of the network components as well as lifetime of the same then only using those parameters one can calculate Fano factor quantity that will provide a hint about the networks fluctuations. This is the main advantage of the present formalism that makes easier the study of stochastic features of several unexplored systems.

In the aforesaid discussion, we have searched for the effect of relaxation time scale on signal transduction machinery through linear type of GTRN motifs. These results suggest us to extend our analysis to motifs having branched pathway. The branched pathway motifs are generated with the help of lateral combinations of more than one linear pathway motifs. Therefore, our next objective is to investigate the relaxation time scale effect on a family of branched network such as feed forward loop (FFL) [2, 12, 33, 38]. FFL appears more frequently in gene networks of *E. coli*, *S. cerevisiae* and other living organisms. It consists of three genes which are characterized by three different transcription factors S, X and Y where X regulates Y and S regulates both X and Y. Thus, S directly as well as indirectly, via X, regulates Y leading to positive (activation) or negative (repression) transcription interaction. Sign of the direct regulation path is equal to the indirect path for coherent feed forward loop (CFFL) but opposite sign of two regulatory pathways is the basis for incoherent feed forward loop (ICFFL). Although eight FFL are possible from structural configuration, we study only two most abundant network motifs, type-1 CFFL and ICFFL. The FFL networks have two input signals, one signal induces the S encoded transcription factor gene and the other induces the X encoded gene. For the sake of simplicity, we investigate these motifs under the effect of constant input signal. In the following subsections, we discuss the role of different time scales on the two FFL motifs.

### Coherent feed forward loop

In type-1 CFFL motif, both S and X either positively or negatively regulate the promoter of the target gene Y. The expression level of output Y is controlled by the concentration of two upstream transcription factors. If both S and X are required to control the production of Y, the motif behaves as AND like logic gate otherwise it behaves as an OR like logic gate. Keeping this in mind, we examine these two architectures and investigate which one of these two faces maximum fluctuations in a noisy environment.

#### OR like gate

In OR like CFFL (OCFFL) motif (Fig 1d), any one of the two upstream components (S and X) is sufficient to control the expression of Y. Hence, in the dynamical equation of Y, we introduce two different synthesis rate constants, $k_{3}^{\prime }$ and *k*_3_ for the direct and the indirect regulation pathways, respectively. These two rate constants give freedom to tune up the extent of interaction amid the transcription factor and the target gene in an independent way. The first term defines the extent of interaction to express Y via direct pathway and the second one is responsible for the indirect pathway. From the logical condition, either of the two interactions of this motif is predominant over the other, i.e., if the direct regulation is stronger then the indirect regulation must be weaker, or vice versa. When the direct regulation path is more prominent, the motif behaves like the OSC but if the indirect path plays a pivotal role, then it performs as the TSC. Keeping this in mind, we write the stochastic dynamical equations as

$$\begin{array}{ccc} \frac{dx}{dt} & = & k_{2}s-\tau_{x}^{-1}x+\xi_{x}\left(t\right), \end{array}$$ (15)

$$\begin{array}{ccc} \frac{dy}{dt} & = & k_{3}^{'}s+k_{3}x-\tau_{y}^{-1}y+\xi_{y}\left(t\right). \end{array}$$ (16)

While writing the above equations, we do not explicitly show the dynamical equation for S component but use the previously written Eq (9) for the OSC. Here the three noise terms (*ξ*_*s*_, *ξ*_*x*_ and *ξ*_*y*_) are of Gaussian white type with zero mean ⟨*ξ*_*s*_(*t*)⟩ = ⟨*ξ*_*x*_(*t*)⟩ = ⟨*ξ*_*y*_(*t*)⟩ = 0. The respective noise strengths are given by $\left\langle \xi_{s}(t)\xi_{s}(t\prime )\rangle =2\tau_{s}^{-1}\langle s\rangle \delta (t-t\prime \right)$, $\left\langle \xi_{x}(t)\xi_{x}(t\prime )\rangle =2\tau_{x}^{-1}\langle x\rangle \delta (t-t\prime \right)$ and $\left\langle \xi_{y}(t)\xi_{y}(t\prime )\rangle =2\tau_{y}^{-1}\langle y\rangle \delta (t-t\prime \right)$, respectively. In addition, the three noise processes are uncorrelated, ⟨*ξ*_*s*_(*t*)*ξ*_*x*_(*t*′)⟩ = ⟨*ξ*_*x*_(*t*)*ξ*_*s*_(*t*′)⟩ = 0, ⟨*ξ*_*x*_(*t*)*ξ*_*y*_(*t*′)⟩ = ⟨*ξ*_*y*_(*t*)*ξ*_*x*_(*t*′)⟩ = 0 and ⟨*ξ*_*s*_(*t*)*ξ*_*y*_(*t*′)⟩ = ⟨*ξ*_*y*_(*t*)*ξ*_*s*_(*t*′)⟩ = 0. Using usual method of solution [42–45, 47, 48], we evaluate the following expression of variance and co-variance

$$\begin{array}{ccc} \sigma_{y}^{2} & = & \left\langle y\right\rangle +\frac{\tau_{y}^{-1}\beta^{2}\left\langle x\right\rangle }{\left(\tau_{x}^{-1}+\tau_{y}^{-1}\right)}+\frac{\tau_{y}^{-1}\gamma^{2}\left\langle s\right\rangle }{\left(\tau_{s}^{-1}+\tau_{y}^{-1}\right)}\\ & & +p\frac{\tau_{x}^{-1}\tau_{y}^{-1}\left(\tau_{s}^{-1}+\tau_{x}^{-1}+\tau_{y}^{-1}\right)\left\langle s\right\rangle }{\left(\tau_{s}^{-1}+\tau_{x}^{-1}\right)\left(\tau_{x}^{-1}+\tau_{y}^{-1}\right)\left(\tau_{s}^{-1}+\tau_{y}^{-1}\right)},\\ \sigma_{sy}^{2} & = & \frac{\tau_{y}^{-1}\left\langle y\right\rangle }{\left(\tau_{s}^{-1}+\tau_{y}^{-1}\right)}-\frac{\tau_{s}^{-1}\tau_{y}^{-1}\beta \left\langle x\right\rangle }{\left(\tau_{s}^{-1}+\tau_{x}^{-1}\right)\left(\tau_{s}^{-1}+\tau_{y}^{-1}\right)}, \end{array}$$ (17)

where *α*, *β*, *γ* and *p* are $k_{2}/\tau_{x}^{-1},k_{3}/\tau_{y}^{-1}$, $k_{3}^{\prime }/\tau_{y}^{-1}$ and *α* ^2^ *β* ^2^ + 2*αβγ*, respectively. All the rate constants define their usual meaning in the above kinetic equations (Eqs (14–15)). To understand the effect of relaxation time scale on this model, we take all possible relations among the three relaxation time constants and obtain nine conditions using which modified expressions of Fano factor and co-variance are calculated (see Table 3).

**Table 3:** Modified forms of the analytical solution (Eq (17)) of OCFFL motif.

		*τ*_*x*_ ≫ *τ*_*y*_	*τ*_*x*_ ≈ *τ*_*y*_	*τ*_*x*_ ≪ *τ*_*y*_
*τ*_*s*_ ≫ *τ*_*x*_	Fano factor	$1+\frac{\beta^{2}\langle x\rangle }{\left\langle y\right\rangle }+\frac{\left(\gamma^{2}+p)\langle s\right\rangle }{\left\langle y\right\rangle }$	$1+0.5\frac{\beta^{2}\langle x\rangle }{\left\langle y\right\rangle }+\frac{\left(\gamma^{2}+p)\langle s\right\rangle }{\left\langle y\right\rangle }$	$1+\frac{\beta^{2}\tau_{x}\langle x\rangle }{\tau_{y}\langle y\rangle }+\rho \frac{\left(\gamma^{2}+p)\langle s\right\rangle }{\left\langle y\right\rangle }$
	$\sigma_{sy}^{2}$	$\langle y\rangle -\frac{\tau_{x}\beta \langle x\rangle }{\tau_{s}}$	$\langle y\rangle -\frac{\tau_{x}\beta \langle x\rangle }{\tau_{s}}$	$\rho (\langle y\rangle -\frac{\tau_{x}\beta \langle x\rangle }{\tau_{s}})$
*τ*_*s*_ ≈ *τ*_*x*_	Fano factor	$1+\frac{\beta^{2}\langle x\rangle }{\left\langle y\right\rangle }+\frac{\left(\gamma^{2}+0.5p)\langle s\right\rangle }{\left\langle y\right\rangle }$	$1+0.5\frac{\beta^{2}\langle x\rangle }{\left\langle y\right\rangle }+\frac{\left({0.5\gamma }^{2}+\frac{3p}{8})\langle s\right\rangle }{\left\langle y\right\rangle }$	$1+\frac{\beta^{2}\tau_{x}\langle x\rangle }{\tau_{y}\langle y\rangle }+\frac{\tau_{s}(\gamma^{2}+p)\langle s\rangle }{\tau_{y}\langle y\rangle }$
	$\sigma_{sy}^{2}$	⟨*y*⟩ − 0.5*β*⟨*x*⟩	0.5⟨*y*⟩ − 0.25*β*⟨*x*⟩	$\frac{\tau_{s}(\langle y\rangle -0.5\beta \langle x\rangle )}{\tau_{y}}$
*τ*_*s*_ ≪ *τ*_*x*_	Fano factor	$1+\frac{\beta^{2}\langle x\rangle }{\left\langle y\right\rangle }+\frac{\left(\rho \gamma^{2}+\frac{\tau_{s}p}{\tau_{x}})\langle s\right\rangle }{\left\langle y\right\rangle }$	$1+0.5\frac{\beta^{2}\langle x\rangle }{\left\langle y\right\rangle }+\frac{\tau_{s}(\gamma^{2}+0.5p)\langle s\rangle }{\tau_{y}\langle y\rangle }$	$1+\frac{\beta^{2}\tau_{x}\langle x\rangle }{\tau_{y}\langle y\rangle }+\frac{\tau_{s}(\gamma^{2}+p)\langle s\rangle }{\tau_{y}\langle y\rangle }$
	$\sigma_{sy}^{2}$	*ρ*(⟨*y*⟩ − *β*⟨*x*⟩)	$\frac{\tau_{s}(\langle y\rangle -\beta \langle x\rangle )}{\tau_{y}}$	$\frac{\tau_{s}(\langle y\rangle -\beta \langle x\rangle )}{\tau_{y}}$

Fano factor ($\sigma_{y}^{2}/\langle y\rangle$) and co-variance ($\sigma_{sy}^{2}$) at different relaxation time limits are shown where *ρ* = *τ*_*s*_/(*τ*_*s*_ + *τ*_*y*_) ⩽ 1, $\alpha =k_{2}/\tau_{x}^{-1}$, $\beta =k_{3}/\tau_{y}^{-1}$, $\gamma =k_{3}^{\prime }/\tau_{y}^{-1}$ and *p* = *α* ^2^ *β* ^2^ + 2*αβγ*.

**Fig 4:**
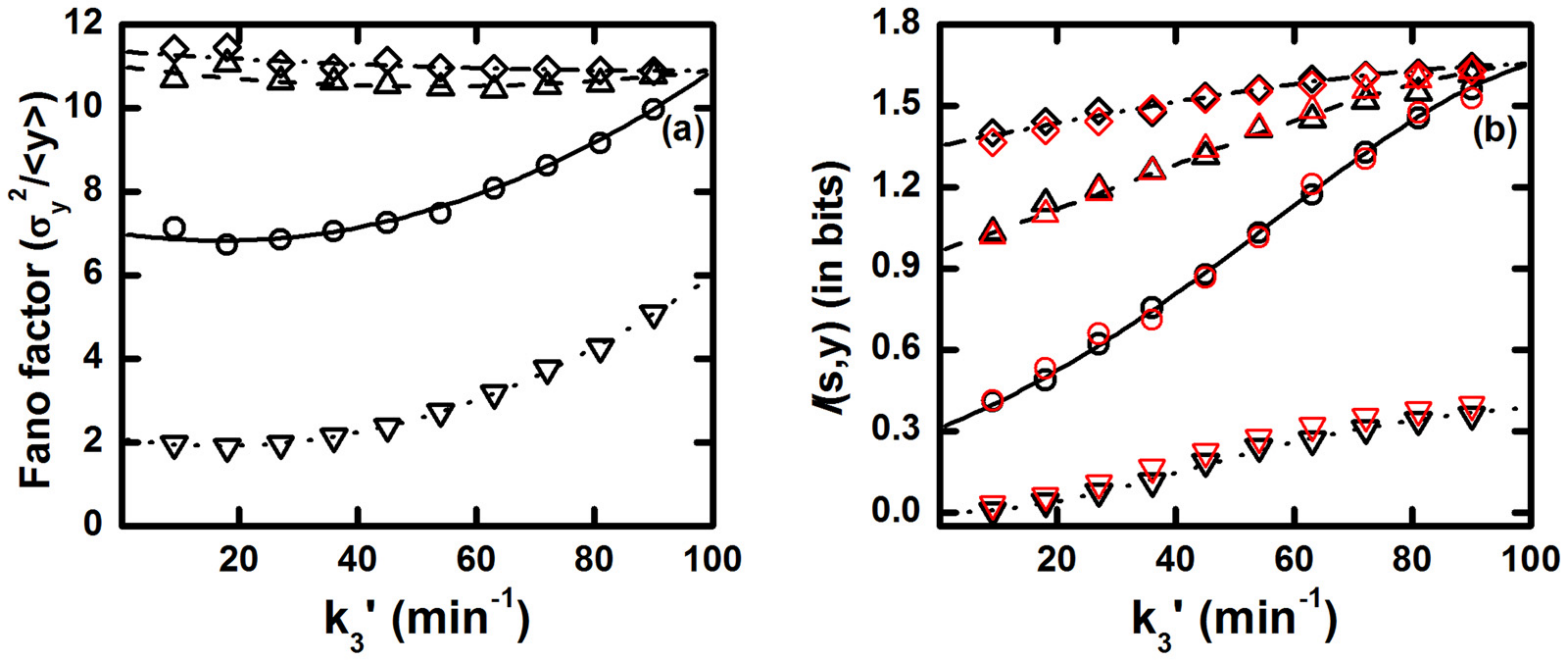
The OCFFL. (a) Fano factor and (b) mutual information 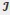
(*s*, *y*) profiles as a function of S dependent synthesis rate constant $k_{3}^{\prime }$ of Y component. The relations $k_{1}/\tau_{s}^{-1}=k_{2}/\tau_{x}^{-1}=10$, $(10k_{3}+k_{3}^{\prime })/\tau_{y}^{-1}=10$ and $\tau_{y}^{-1}=10$ min^−1^ are maintained so that steady state population of all the components remain unaltered. In both plots, for solid (with open circles), dashed (with open upward triangle), dotted (with open downward triangle) and dash dotted (with open diamond) lines we have used $\tau_{s}^{-1}=\tau_{x}^{-1}=0.1$ min^−1^, $\tau_{s}^{-1}=\tau_{x}^{-1}/10=0.1$ min^−1^, $\tau_{s}^{-1}/100=\tau_{x}^{-1}=0.1$ min^−1^ and $\tau_{s}^{-1}=\tau_{x}^{-1}/100=0.1$ min^−1^, respectively. The symbols are generated using stochastic simulation algorithm [64, 65] and the lines are due to theoretical calculation. In the plot of mutual information the red and black symbols are due to Eq (7) and Eq (8), respectively.

Similar to our previously discussed motifs, the OCFFL motif also accomplishes maximum, intermediate and minimum values of Fano factor and co-variance for the three separate relaxation time limiting conditions *τ*_*s*_ ≫ *τ*_*x*_ ≫ *τ*_*y*_, *τ*_*s*_ ≈ *τ*_*x*_ ≈ *τ*_*y*_ and *τ*_*s*_ ≪ *τ*_*x*_ ≪ *τ*_*y*_. These modified (maximum, intermediate and minimum) forms are given in Table 3. For OCFFL, we do not explore graphically the role of relaxation rate constant $\tau_{y}^{-1}$ on Fano factor and mutual information. Due to the presence of an interesting feature in this motif by the virtue of direct and indirect contribution of two regulatory pathways in gene regulation via S and X, respectively, we look at the dependence of the rate constants $k_{3}^{\prime }$ and *k*_3_. Since, on the basis of the dominating power amid the two rate constants, the OCFFL motif can reduce to either OSC or TSC, we investigate the effect of these synthesis rate constants on the fluctuations and mutual information propagation. We show Fano factor and mutual information 𝓘(*s*, *y*) as a function of $k_{3}^{\prime }$ in Fig 4. For both plots, we maintain a constant pool of the steady state population level of the Y component using the relation $(10k_{3}+k_{3}^{\prime })/\tau_{y}^{-1}=10$ and set a high value of the relaxation rate constant $\tau_{y}^{-1}=10$ min^−1^. Only in this relaxation time domain, the output component can track fluctuations in the upstream signal very accurately.

In Fig 4, we increase the influence of direct regulatory pathway with the help of augmentation of the S dependent synthesis rate constant $k_{3}^{\prime }$ of the Y component. By doing this, we increase the contribution of the OSC motif in the OCFFL but decrease the contribution of X dependent synthesis rate constant *k*_3_ due to the relation $(10k_{3}+k_{3}^{\prime })/\tau_{y}^{-1}=10$. In Fig 4a, Fano factor profile slowly goes down with $k_{3}^{\prime }$ for $\tau_{s}^{-1}=\tau_{x}^{-1}/10=0.1$ min^−1^ and $\tau_{s}^{-1}=\tau_{x}^{-1}/100=0.1$ min^−1^. At these two limits, fluctuations can propagate through this motif very smoothly. For very low value of $k_{3}^{\prime }$, the OCFFL motif behaves as a TSC with high Fano factor value. On the other hand, for high value of $k_{3}^{\prime }$, the OCFFL motif behaves as a OSC with comparatively low Fano factor value. For the rest of the two parameter sets ($\tau_{s}^{-1}=\tau_{x}^{-1}=0.1$ min^−1^ and $\tau_{s}^{-1}/100=\tau_{x}^{-1}=0.1$ min^−1^), the Fano factor value increases with $k_{3}^{\prime }$ as fluctuations propagation is hindered by the intermediate component X due to the indirect pathway. At these limits, for low value of $k_{3}^{\prime }$, the motif attains a lower Fano factor value but as the value of $k_{3}^{\prime }$ increases, the extent of OSC character plays a dominant role which in turn increases the Fano factor value and fluctuations propagation becomes smooth via the direct pathway without any sort of intermediate obstacle.

In Fig 4b, we show the mutual information 𝓘(*s*, *y*) in between the input signal S and the output Y as a function of $k_{3}^{\prime }$. The profile shows an increasing trend and is valid for all sets of parameter considered. Information processing is mainly affected by fluctuations and number of intermediate component(s) in between the input and the output of the corresponding network. Hence, at low value of $k_{3}^{\prime }$, lesser amount of signal is transmitted compared to high value of $k_{3}^{\prime }$ due to the transition from effective TSC character to effective OSC character as $k_{3}^{\prime }$ is increased. Among the four parameter sets considered, exceptionally low 𝓘(*s*, *y*) value is obtained for $\tau_{s}^{-1}/100=\tau_{x}^{-1}=0.1$ min^−1^ due to faster fluctuations rate of S component than X and Y components. Living systems having the OCFFL motif have a great advantage of adopting either of the two linear cascades (OSC or TSC) with the variation of weightage on direct or indirect pathway of gene regulation. Thus, any system when gives an extra importance on the TSC, attains maximum output fluctuations with minimum mutual information but attains reverse results by giving importance to the OSC motif. A trade off between output fluctuations and mutual information may be accomplished by the system using these two pathways, direct and indirect. Therefore, the essence of this motif is that during evolution it has been designed in a way that makes a living system more adaptable within any diverse environmental situation.

#### AND like gate

In the AND like CFFL (ACFFL) motif (Fig 1e), both S and X jointly regulate the target gene Y. Thus, in the dynamical equation of Y, the synthesis term is expressed in terms of both S and X. Other than this synthesis part, rest of the equations of all the dynamical components are the same as the OCFFL motif,

$$\begin{array}{ccc} \frac{dx}{dt} & = & k_{2}s-\tau_{x}^{-1}x+\xi_{x}\left(t\right), \end{array}$$ (18)

$$\begin{array}{ccc} \frac{dy}{dt} & = & k_{3}sx-\tau_{y}^{-1}y+\xi_{y}\left(t\right). \end{array}$$ (19)

In the above set of equations, all the rate constants define kinetic significance of the corresponding network components. Here, we also do not rewrite the dynamical equation for S and use the previous equation (Eq (9)). Here *ξ*_*s*_, *ξ*_*x*_ and *ξ*_*y*_ are Gaussian white noise with zero mean ⟨*ξ*_*s*_(*t*)⟩ = ⟨*ξ*_*x*_(*t*)⟩ = ⟨*ξ*_*y*_(*t*)⟩ = 0. The respective noise strengths are given by $\left\langle \xi_{s}(t)\xi_{s}(t\prime )\rangle =2\tau_{s}^{-1}\langle s\rangle \delta (t-t\prime \right)$, $\left\langle \xi_{x}(t)\xi_{x}(t\prime )\rangle =2\tau_{x}^{-1}\langle x\rangle \delta (t-t\prime \right)$ and $\left\langle \xi_{y}(t)\xi_{y}(t\prime )\rangle =2\tau_{y}^{-1}\langle y\rangle \delta (t-t\prime \right)$, respectively. In addition, the three noise processes are uncorrelated, ⟨*ξ*_*s*_(*t*)*ξ*_*x*_(*t*′)⟩ = ⟨*ξ*_*x*_(*t*)*ξ*_*s*_(*t*′)⟩ = 0, ⟨*ξ*_*x*_(*t*)*ξ*_*y*_(*t*′)⟩ = ⟨*ξ*_*y*_(*t*)*ξ*_*x*_(*t*′)⟩ = 0 and ⟨*ξ*_*s*_(*t*)*ξ*_*y*_(*t*′)⟩ = ⟨*ξ*_*y*_(*t*)*ξ*_*s*_(*t*′)⟩ = 0. We solve the set of dynamical equations considering an approximation ⟨*sx*⟩ = ⟨*s*⟩⟨*x*⟩ [42–45, 47, 48] as reactions in between the two components S and X are uncorrelated with each other and obtain simplified analytical form of variance and co-variance of the target gene Y as

$$\begin{array}{ccc} \sigma_{y}^{2} & = & \left\langle y\right\rangle +\frac{\tau_{y}^{-1}{\left\langle y\right\rangle }^{2}}{\left(\tau_{x}^{-1}+\tau_{y}^{-1}\right)\left\langle x\right\rangle }+\frac{\tau_{y}^{-1}{\left\langle y\right\rangle }^{2}}{\left(\tau_{s}^{-1}+\tau_{y}^{-1}\right)\left\langle s\right\rangle }\\ & & +\frac{3\tau_{x}^{-1}\tau_{y}^{-1}\left(\tau_{s}^{-1}+\tau_{x}^{-1}+\tau_{y}^{-1}\right){\left\langle y\right\rangle }^{2}}{\left(\tau_{s}^{-1}+\tau_{x}^{-1}\right)\left(\tau_{x}^{-1}+\tau_{y}^{-1}\right)\left(\tau_{s}^{-1}+\tau_{y}^{-1}\right)\left\langle s\right\rangle },\\ \sigma_{sy}^{2} & = & \frac{\tau_{y}^{-1}\left\langle y\right\rangle }{\left(\tau_{s}^{-1}+\tau_{y}^{-1}\right)}+\frac{\tau_{x}^{-1}\tau_{y}^{-1}\left\langle y\right\rangle }{\left(\tau_{s}^{-1}+\tau_{x}^{-1}\right)\left(\tau_{s}^{-1}+\tau_{y}^{-1}\right)}. \end{array}$$ (20)

It is interesting to note that for both TSC and ACFFL, we get almost equivalent variance expression (see Eq (12) and Eq (19)) except for two factors. These extra terms are the third and the fourth term on the right hand side of Eq (19). The third term arises due to direct regulation of Y by S. The fourth term is multiplied by a numerical factor 3. From these extra terms, it is obvious that ACFFL shows higher fluctuating property than TSC due to the additive nature of two positive regulatory pathways (both direct and indirect) [2, 21, 78–80]. From Eq (19), we get all possible reduced forms of Fano factor and co-variance expressions using nine possible relations among the three relaxation time scales (see Table 4).

**Table 4:** Modified forms of the analytical solution (Eq 18) of ACFFL motif.

		*τ*_*x*_ ≫ *τ*_*y*_	*τ*_*x*_ ≈ *τ*_*y*_	*τ*_*x*_ ≪ *τ*_*y*_
*τ*_*s*_ ≫ *τ*_*x*_	Fano factor	$1+\frac{\left\langle y\right\rangle }{\left\langle x\right\rangle }+4\frac{\left\langle y\right\rangle }{\left\langle s\right\rangle }$	$1+0.5\frac{\left\langle y\right\rangle }{\left\langle x\right\rangle }+4\frac{\left\langle y\right\rangle }{\left\langle s\right\rangle }$	$1+\frac{\tau_{x}\langle y\rangle }{\tau_{y}\langle x\rangle }+4\rho \frac{\left\langle y\right\rangle }{\left\langle s\right\rangle }$
	$\sigma_{sy}^{2}$	2⟨*y*⟩	2⟨*y*⟩	2*ρ*⟨*y*⟩
*τ*_*s*_ ≈ *τ*_*x*_	Fano factor	$1+\frac{\left\langle y\right\rangle }{\left\langle x\right\rangle }+2.5\frac{\left\langle y\right\rangle }{\left\langle s\right\rangle }$	$1+0.5\frac{\left\langle y\right\rangle }{\left\langle x\right\rangle }+\frac{13\langle y\rangle }{8\langle s\rangle }$	$1+\frac{\tau_{x}\langle y\rangle }{\tau_{y}\langle x\rangle }+4\frac{\tau_{x}\langle y\rangle }{\tau_{y}\langle s\rangle }$
	$\sigma_{sy}^{2}$	1.5⟨*y*⟩	0.75⟨*y*⟩	$1.5\frac{\tau_{s}\langle y\rangle }{\tau_{y}}$
*τ*_*s*_ ≪ *τ*_*x*_	Fano factor	$1+\frac{\left\langle y\right\rangle }{\left\langle x\right\rangle }+\frac{\left(\rho +3\frac{\tau_{s}}{\tau_{x}})\langle y\right\rangle }{\left\langle x\right\rangle }$	$1+0.5\frac{\left\langle y\right\rangle }{\left\langle x\right\rangle }+2.5\frac{\tau_{s}\langle y\rangle }{\tau_{x}\langle s\rangle }$	$1+\frac{\tau_{x}\langle y\rangle }{\tau_{y}\langle x\rangle }+4\frac{\tau_{s}\langle y\rangle }{\tau_{y}\langle s\rangle }$
	$\sigma_{sy}^{2}$	$\left(1+\frac{\tau_{s}}{\tau_{x}})\rho \langle y\right\rangle$	$(1+\frac{\tau_{s}}{\tau_{x}})\frac{\tau_{s}\langle y\rangle }{\tau_{y}}$	$(1+\frac{\tau_{s}}{\tau_{x}})\frac{\tau_{s}\langle y\rangle }{\tau_{y}}$

Fano factor ($\sigma_{y}^{2}/\langle y\rangle$) and co-variance ($\sigma_{sy}^{2}$) at different relaxation time limits are shown where *ρ* = *τ*_*s*_/(*τ*_*s*_ + *τ*_*y*_) ⩽ 1.

**Fig 5:**
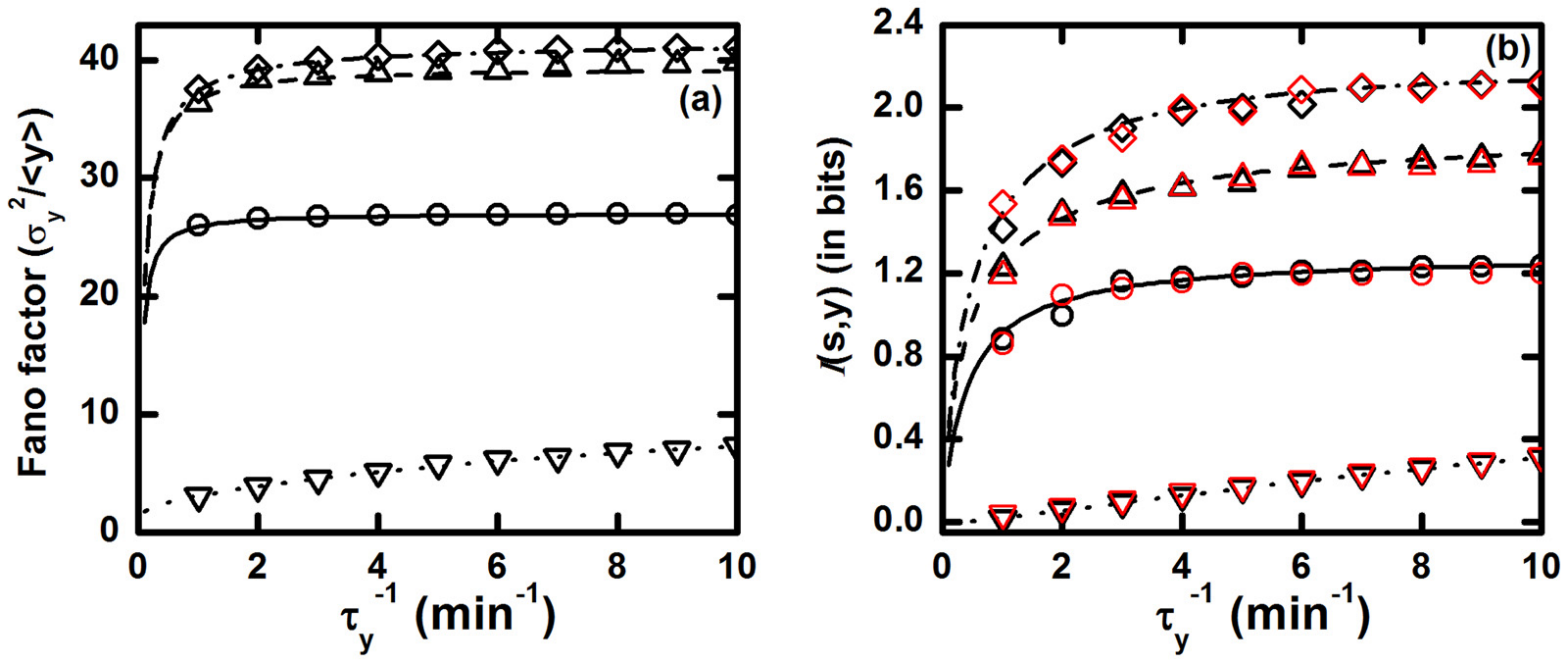
The ACFFL. (a) Fano factor and (b) mutual information 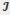
(*s*, *y*) profiles as a function of relaxation rate constant $\tau_{y}^{-1}$ of Y component. The relations $k_{1}/\tau_{s}^{-1}=k_{2}/\tau_{x}^{-1}=10$ and $k_{3}/\tau_{y}^{-1}=0.1$ are maintained so that steady state population of all the components remain unaltered. In both plots, for solid (with open circles), dashed (with open upward triangle), doted (with open downward triangle) and dash dotted (with open diamond) lines we have used $\tau_{s}^{-1}=\tau_{x}^{-1}=0.1$ min^−1^, $\tau_{s}^{-1}=\tau_{x}^{-1}/10=0.1$ min^−1^, $\tau_{s}^{-1}/100=\tau_{x}^{-1}=0.1$ min^−1^ and $\tau_{s}^{-1}=\tau_{x}^{-1}/100=0.1$ min^−1^, respectively. The symbols are generated using stochastic simulation algorithm [64, 65] and the lines are due to theoretical calculation. In the plot of mutual information the red and black symbols are due to Eq (7) and Eq (8), respectively.

As shown in the calculation for previous motifs, it is clear from Table 4 that, for ACFFL, Fano factor and co-variance achieve maximum, intermediate and minimum values for *τ*_*s*_ ≫ *τ*_*x*_ ≫ *τ*_*y*_, *τ*_*s*_ ≈ *τ*_*x*_ ≈ *τ*_*y*_ and *τ*_*s*_ ≪ *τ*_*x*_ ≪ *τ*_*y*_, respectively. At these time scales, the modified forms of both Fano factor and co-variance are almost similar with the modified forms of TSC (see Table 2) but terms like ⟨*y*⟩/⟨*s*⟩ and ⟨*y*⟩ appear with multiplicative factor greater than 1. This leads to a high level of fluctuating environment for the ACFFL motif. The main reason behind the elevation of output fluctuations is the addition of fluctuations due to input signal S into the total fluctuations of the output component Y in two ways, direct and indirect pathways. Due to the such types of fluctuations addition phenomena, we get a higher Fano factor value. Similarly, we also obtain high level of mutual information 𝓘(*s*, *y*) transduction due to the presence of two subsequent pathways by which the target gene reliably accumulates signal information with greater extent and transcribes gene products precisely with the variation of input signal. To verify these features, we show Fano factor and mutual information as a function of relaxation rate constant $\tau_{y}^{-1}$ for four different parameter sets of relaxation rate constants in Fig 5.

In Fig 5a, highest Fano factor value is obtained for the parameter sets $\tau_{s}^{-1}=\tau_{x}^{-1}/10=0.1$ min^−1^ and $\tau_{s}^{-1}=\tau_{x}^{-1}/100=0.1$ min^−1^. Both sets are due to faster fluctuations of the intermediate component X. On the other hand, lowest Fano factor value is attained for $\tau_{s}^{-1}/100=\tau_{x}^{-1}=0.1$ min^−1^ and is due to faster fluctuations of input signal S. For $\tau_{s}^{-1}=\tau_{x}^{-1}=0.1$ min^−1^, the motif shows an intermediate Fano factor value. In all cases, Fano factor value increases with the relaxation rate constant of the target gene Y. Similar trend is also observed for the mutual information 𝓘(*s*, *y*) profile (Fig 5b). Therefore, this motif has high information processing capacity in spite of the presence of high level of fluctuations. Such high input signal processing phenomena facilitate the networks reliability for signal transduction in GTRN and makes this motif a highly abundant one among rest of the CFFL present within the family of cellular networks. In this connection, it is important to mention that the network architecture can facilitate cellular fitness advantage in adverse environment due to increase of fluctuations. Thus, high fluctuations in output gene expression trigger phenotypic heterogeneity in clonal cell populations and can induce drug resistance [26, 81]. A similar type of CFFL is also liable for drug resistance of human cancer cells.

### Incoherent feed forward loop

The last motif considered in the present work is another class of FFL known as Incoherent feed forward loop. We focus only on the type-1 incoherent feed forward loop (ICFFL) (Fig 1f). In this motif, two regulatory pathways act in an opposite manner. Here, input signal S positively regulates the target gene Y through the direct pathway. However, the intermediate component X represses the expression of Y. As a result, S initially activates both X and Y rapidly but after some time, population level of X reaches a threshold to repress the production of Y. While modeling the repression phenomenon, we consider the Hill coefficient to be one. Thus, the Langevin equations for the dynamical quantities can be written as

$$\begin{array}{ccc} \frac{dx}{dt} & = & k_{2}s-\tau_{x}^{-1}x+\xi_{x}\left(t\right), \end{array}$$ (21)

$$\begin{array}{ccc} \frac{dy}{dt} & = & k_{3}g\left(x\right)s-\tau_{y}^{-1}y+\xi_{y}\left(t\right). \end{array}$$ (22)

In the above equation, *g*(*x*) = *K*/(*K* + *x*) is a nonlinear repressive function that depends on the concentration of X and *K*, the ratio of unbinding to binding rate constants of transcription factor X at the promoter region of Y gene. Here we consider *K* = 1. In addition, we also consider ⟨*g*(*x*)*s*⟩ = ⟨*g*(*x*)⟩⟨*s*⟩ as the kinetic equations for both S and X are uncorrelated with each other and ⟨*x*⟩/(*K* + ⟨*x*⟩) ≈ 1 as steady state concentration of the X component is much higher than the unbinding-binding constant (*x* ≫ *K*). For this motif, we use the earlier kinetic equation for S (see Eq (9)). Here *ξ*_*s*_, *ξ*_*x*_ and *ξ*_*y*_ are Gaussian white noise with zero mean ⟨*ξ*_*s*_(*t*)⟩ = ⟨*ξ*_*x*_(*t*)⟩ = ⟨*ξ*_*y*_(*t*)⟩ = 0. The respective noise strengths are given by $\left\langle \xi_{s}(t)\xi_{s}(t\prime )\rangle =2\tau_{s}^{-1}\langle s\rangle \delta (t-t\prime \right)$, $\left\langle \xi_{x}(t)\xi_{x}(t\prime )\rangle =2\tau_{x}^{-1}\langle x\rangle \delta (t-t\prime \right)$ and $\left\langle \xi_{y}(t)\xi_{y}(t\prime )\rangle =2\tau_{y}^{-1}\langle y\rangle \delta (t-t\prime \right)$, respectively. In addition, the three noise processes are uncorrelated, ⟨*ξ*_*s*_(*t*)*ξ*_*x*_(*t*′)⟩ = ⟨*ξ*_*x*_(*t*)*ξ*_*s*_(*t*′)⟩ = 0, ⟨*ξ*_*x*_(*t*)*ξ*_*y*_(*t*′)⟩ = ⟨*ξ*_*y*_(*t*)*ξ*_*x*_(*t*′)⟩ = 0 and ⟨*ξ*_*s*_(*t*)*ξ*_*y*_(*t*′)⟩ = ⟨*ξ*_*y*_(*t*)*ξ*_*s*_(*t*′)⟩ = 0. Solving the set of kinetic equations, we get the simplified mathematical form of variance and co-variance [42–45, 47, 48]

$$\begin{array}{ccc} \sigma_{y}^{2} & = & \left\langle y\right\rangle +\frac{\tau_{y}^{-1}{\left\langle y\right\rangle }^{2}}{\left(\tau_{x}^{-1}+\tau_{y}^{-1}\right)\left\langle x\right\rangle }+\frac{\tau_{y}^{-1}{\left\langle y\right\rangle }^{2}}{\left(\tau_{s}^{-1}+\tau_{y}^{-1}\right)\left\langle s\right\rangle }\\ & & -\frac{\tau_{x}^{-1}\tau_{y}^{-1}\left(\tau_{s}^{-1}+\tau_{x}^{-1}+\tau_{y}^{-1}\right){\left\langle y\right\rangle }^{2}}{\left(\tau_{s}^{-1}+\tau_{x}^{-1}\right)\left(\tau_{x}^{-1}+\tau_{y}^{-1}\right)\left(\tau_{s}^{-1}+\tau_{y}^{-1}\right)\left\langle s\right\rangle },\\ \sigma_{sy}^{2} & = & \frac{\tau_{y}^{-1}\left\langle y\right\rangle }{\left(\tau_{s}^{-1}+\tau_{y}^{-1}\right)}-\frac{\tau_{x}^{-1}\tau_{y}^{-1}\left\langle y\right\rangle }{\left(\tau_{s}^{-1}+\tau_{x}^{-1}\right)\left(\tau_{s}^{-1}+\tau_{y}^{-1}\right)}. \end{array}$$ (23)

In the above variance expression, two S dependent fluctuations terms are present with opposite sign thus compensating each other. The variance expression is quite similar to the expression of TSC (see Eq (12)) but differs in the sign of the last term and the appearance of an extra term due to direct regulation of Y by S. If one calculates the magnitude of variance for both TSC and ICFFL using unique steady state population level of all three components and the corresponding relaxation time scale, a higher value of $\sigma_{y}^{2}$ will be observed for TSC compared to ICFFL. To check the validity of this effect, we consider all possible relations among the three relaxation times of the corresponding network components and get nine possible relations. Using these relations, modified forms of Fano factor and co-variance are calculated and are given in Table 5. From these modified expressions, it is clear that ICFFL exerts lesser amount of fluctuations compared to the other motifs considered in this work while developing the target gene product Y. Development of low fluctuations thus gets reflected in the Fano factor value. This happens due to repression of target gene by X which effectively reduces fluctuations associated with Y [2, 21, 78–80]. It is important to note that, in Table 5, under some relaxation time scale limits, the Fano factor expressions are free from terms with ⟨*s*⟩, the S dependent fluctuations. This happens due to a simultaneous contribution of direct and indirect pathways which cancel each other by having equal magnitude but opposite sign. As a consequence, some of the co-variance values also become zero. This suggests that at these time scale limits, the system cannot incorporate the information of the input signal properly, thereby transducing information about the input signal unreliably to the target gene. Keeping this in mind, we explore the nature of Fano factor and mutual information 𝓘(*s*, *y*) as a function of relaxation rate constant $\tau_{y}^{-1}$ of Y component using four different sets, i.e., $\tau_{s}^{-1}=\tau_{x}^{-1}=0.1$ min^−1^, $\tau_{s}^{-1}=\tau_{x}^{-1}/10=0.1$ min^−1^, $\tau_{s}^{-1}/100=\tau_{x}^{-1}=0.1$ min^−1^ and $\tau_{s}^{-1}=\tau_{x}^{-1}/100=0.1$ min^−1^.

**Table 5:** Modified form of the analytical solution (Eq (23)) of ICFFL motif.

		*τ*_*x*_ ≫ *τ*_*y*_	*τ*_*x*_ ≈ *τ*_*y*_	*τ*_*x*_ ≪ *τ*_*y*_
*τ*_*s*_ ≫ *τ*_*x*_	Fano factor	$1+\frac{\left\langle y\right\rangle }{\left\langle x\right\rangle }$	$1+0.5\frac{\left\langle y\right\rangle }{\left\langle x\right\rangle }$	$1+\frac{\tau_{x}\langle y\rangle }{\tau_{y}\langle x\rangle }$
	$\sigma_{sy}^{2}$	0	0	0
*τ*_*s*_ ≈ *τ*_*x*_	Fano factor	$1+\frac{\left\langle y\right\rangle }{\left\langle x\right\rangle }+0.5\frac{\left\langle y\right\rangle }{\left\langle s\right\rangle }$	$1+0.5\frac{\left\langle y\right\rangle }{\left\langle x\right\rangle }+\frac{\left\langle y\right\rangle }{8\langle s\rangle }$	$1+\frac{\tau_{x}\langle y\rangle }{\tau_{y}\langle x\rangle }$
	$\sigma_{sy}^{2}$	0.5⟨*y*⟩	0.25⟨*y*⟩	$0.5\frac{\tau_{s}\langle y\rangle }{\tau_{y}}$
*τ*_*s*_ ≪ *τ*_*x*_	Fano factor	$1+\frac{\left\langle y\right\rangle }{\left\langle x\right\rangle }+\frac{\left(\rho -\frac{\tau_{s}}{\tau_{x}})\langle y\right\rangle }{\left\langle s\right\rangle }$	$1+0.5\frac{\left\langle y\right\rangle }{\left\langle x\right\rangle }+0.5\frac{\tau_{s}\langle y\rangle }{\tau_{y}\langle s\rangle }$	$1+\frac{\tau_{x}\langle y\rangle }{\tau_{y}\langle x\rangle }$
	$\sigma_{sy}^{2}$	$\left(1-\frac{\tau_{s}}{\tau_{x}})\rho \langle y\right\rangle$	$(1-\frac{\tau_{s}}{\tau_{x}})\frac{\tau_{s}\langle y\rangle }{\tau_{y}}$	$(1-\frac{\tau_{s}}{\tau_{x}})\frac{\tau_{s}\langle y\rangle }{\tau_{y}}$

Fano factor ($\sigma_{y}^{2}/\langle y\rangle$) and co-variance ($\sigma_{sy}^{2}$) at different relaxation time limits are shown where *ρ* = *τ*_*s*_/(*τ*_*s*_ + *τ*_*y*_) ⩽ 1.

**Fig 6:**
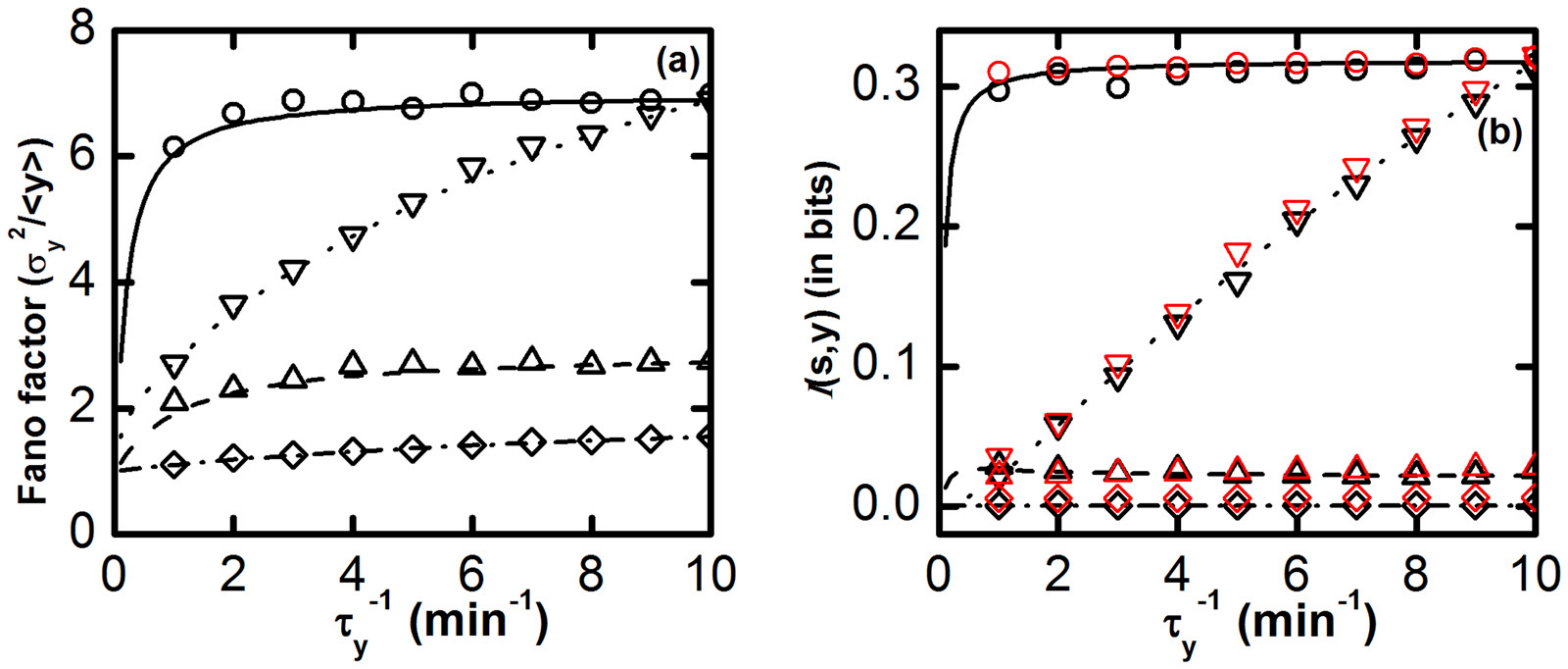
The ICFFL. (a) Fano factor and (b) mutual information 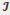
(*s*, *y*) profiles as function of relaxation rate constant $\tau_{y}^{-1}$ of Y component. $k_{1}/\tau_{s}^{-1}=k_{2}/\tau_{x}^{-1}=10$ and $k_{3}/\tau_{y}^{-1}=1010$, ratios are maintained throughout these plots, so steady state population of all the components remain unaltered. In both plots, for solid (with open circles), dash (with open upward triangle), dot (with open downward triangle) and dash dot (with open diamond) lines, we use the following sets of parameters: $\tau_{s}^{-1}=\tau_{x}^{-1}=0.1$ min^−1^, $\tau_{s}^{-1}=\tau_{x}^{-1}/10=0.1$ min^−1^, $\tau_{s}^{-1}/100=\tau_{x}^{-1}=0.1$ min^−1^ and $\tau_{s}^{-1}=\tau_{x}^{-1}/100=0.1$ min^−1^, respectively. *K* = 1. The symbols are generated using stochastic simulation algorithm and the lines are from theoretical calculation. The symbols are generated using stochastic simulation algorithm [64, 65] and the lines are due to theoretical calculation. In the plot of mutual information the red and black symbols are due to Eq (7) and Eq (8), respectively.

In Fig 6a, Fano factor value gradually increases with the relaxation rate constant $\tau_{y}^{-1}$. Very low level of Fano factor value is attained by the motif for $\tau_{s}^{-1}=\tau_{x}^{-1}/10=0.1$ min^−1^ and $\tau_{s}^{-1}=\tau_{x}^{-1}/100=0.1$ min^−1^. For these two sets, input fluctuations flow successfully through the direct and indirect pathways. Thus, two S dependent fluctuating terms that are equal in magnitude but opposite in sign compensate each other, consequently suppressing the fluctuations associated with the network. On the other hand, for the other two parameter sets, a high level of Fano factor values is found. This happens due to slower rate of fluctuations in the intermediate X. The input fluctuations that come through the indirect pathway are filtered out by X for its low pass filter nature. As a result, two S dependent fluctuating terms do not completely cancel each other. In these parameter sets, the fluctuating part that contributes to the direct pathway shows its prominent effect than the fluctuations due to indirect pathway which finally gets reflected in the total output fluctuations. Likewise, in Fig 6b, mutual information 𝓘(*s*, *y*) values are near to zero for the first two parameter sets giving low level of Fano factor [56] and significant 𝓘(*s*, *y*) values for the rest of the parameter sets.

## Conclusions

In this paper, we have analyzed transmission of a fluctuating input signal through some biochemical signaling networks. We have analytically calculated Fano factor associated with the output and mutual information between the input and the output for two linear and three branched motifs to comprehend the significance of these networks in biological systems. On the basis of linear noise approximation, we have solved linear and nonlinear Langevin equations and verified the analytical results with exact stochastic simulation data of the corresponding networks and found that the approximation method is quite accurate as proposed earlier [48, 59]. In the analytical calculation, we have considered that all noise terms (*ξ*_*s*_, *ξ*_*x*_ and *ξ*_*y*_) are Gaussian in nature and the effect of cross correlation between two noise terms is zero. We have calculated Fano factor and mutual information for five motifs with the variation of relaxation rate constants associated with all network components and have studied effect of input signal on these two measurable quantities. Our study not only takes care of an individual motif but also presents a comparative study of all the biochemical motifs in the light of Fano factor and mutual information. For graphical presentation of the output of each motif, we have tuned synthesis and relaxation rate constants of each network component in such a way so that the steady state population of the same remains constant. In addition, the aforesaid strategy preserves the total population of the component in each motif. Adoption of such a strategy helps us to apprehend how Fano factor and mutual information values get affected from one motif to another under equal population of network component.

We have started our calculations considering linear type of cascades. The first motif we have considered is OSC where the motif can precisely characterize the information of input signal at faster relaxation time of the output component compared to the input one. This accuracy gradually decreases with the increment of input relaxation rate. We have also shown that the OSC motif is unable to differentiate the variation of input signal at high population limit of the input component. We then compare this motif with the standard gene regulation network to explicate such time scale effect on gene regulation and found some significant circumstances in which the gene regulatory network can tune up optimum fluctuations for both essential and nonessential proteins. Our analysis is at par with the results of Fraser et al [75] where they have performed analysis using several experimentally determined gene regulation rates.

The second motif considered is TSC where an intermediate component is present between the components of OSC. Through our analysis, we have observed that Fano factor of output gets amplified in magnitude and is in agreement with the analysis provided by Bowsher et al [37]. In addition, similar kind of relaxation time scale effect for propagation of fluctuations is also present in this motif, as observed in OSC. Our analysis suggests that relaxation time scale of the intermediate component is a crucial factor for signal transmission in this motif and can control fluctuations associated with the output. The intermediate component acts as a low pass filter for very fast fluctuations and hinders input fluctuations that flow through the TSC motif. After analyzing the TSC motif, we have introduced *n* number of intermediate components in between the input and the output component to generate a generalized linear long chain cascade and derived simplified form of Fano factor expression for three distinct time scale limits. We have shown that the output fluctuations increase with number of intermediate components, which is in agreement with previously published experimental and theoretical results. The main utility of these expressions is that one can easily calculate fluctuations associated with any linear long chain cascade without enough knowledge of the parameter values of the network.

Next, we have chosen FFL, a group of branched pathways that are abundant in the signal transduction machinery of living systems and are generated by lateral combination of OSC and TSC with different modes of interaction. At first, we have studied OCFFL from FFL group and calculated Fano factor and mutual information with the extent of direct (OSC) and indirect (TSC) contribution of input signal to the target gene. In our calculation of Fano factor, we have observed two opposing behavior while tuning the control parameter. In one case, Fano factor decreases slowly while in the other situation it shows an increasing trend. These results together suggest that OCFFL performs differently for diverse relaxation time scale limits. Furthermore, depending on the contribution of the direct and the indirect pathway, it can acquire the character of OSC and TCF motif, respectively. Such quality of OCFFL helps a living system to survive in diverse environmental conditions. We then extend our analysis to ACFFL where a high value of Fano factor and mutual information is observed. Such high values of Fano factor and mutual information reveal that ACFFL motif can transduce the signal with high reliability and supports its ubiquitous presence in several biological species. The last motif we have considered is ICFFL where output fluctuations are suppressed as the target gene is simultaneously regulated negatively (via indirect pathway with TSC character) as well as positively (via direct pathway with OSC character) by the input signal. This leads to a very low value of Fano factor [56] and mutual information for ICFFL motif. Such low value suggests that living system containing this motif faces minimum fluctuations with low mutual information propagation.

To conclude, we emphasize that our methodology is a general one and is applicable for studying the dynamics of other network motifs under single cell environment. At this point, it is important to mention that enough single cell data are not available for FFL. Our theoretical results thus can act as a starting point to experimentally verify the stochastic dynamics of the network motifs. The results we have derived in the present work can be tested by performing experiment at a single cell level.

## Supporting Information

S1 Table Tables for chemical reactions, propensity function and rate constant for different motifs. (PDF)
