# Supplementary material for: Role of Relaxation Time Scale in Noisy Signal Transduction
Source: PLoS One. 2015 May 8;10(5):e0123242. doi: 10.1371/journal.pone.0123242 (PMC4425683; doi:10.1371/journal.pone.0123242)
Supplement: S1 Table — (PDF) [file pone.0123242.s001.pdf]

Role of relaxation time scale in noisy signal transduction

Alok Kumar Maity, Pinaki Chaudhury and Suman K Banik

Tables for chemical reactions, propensity function and rate constant for different motifs. In all the tables unit of s, x and y are expressed in molecules/V where V is the unit cellular volume. As a result, rate constants are expressed using standard convention [1].

Supporting Table I: OSC motif.

Description	Reaction	Propensity function	Rate constant (min^{-1})
Synthesis of S	$\phi \longrightarrow$ S	k_1	k_1
Degradation of S	S $\longrightarrow \phi$	$\tau_s^{-1} s$	τ_s^{-1}
S mediated synthesis of Y	S $\longrightarrow$ S+Y	$k_3 s$	k_3
Degradation of Y	Y $\longrightarrow \phi$	$\tau_y^{-1} y$	τ_y^{-1}

Supporting Table II: TSC motif.

Description	Reaction	Propensity function	Rate constant (min^{-1})
Synthesis of S	$\phi \longrightarrow$ S	k_1	k_1
Degradation of S	S $\longrightarrow \phi$	$\tau_s^{-1} s$	τ_s^{-1}
S mediated synthesis of X	S $\longrightarrow$ S+X	$k_2 s$	k_2
Degradation of X	X $\longrightarrow \phi$	$\tau_x^{-1} x$	τ_x^{-1}
X mediated synthesis of Y	X $\longrightarrow$ X+Y	$k_3 x$	k_3
Degradation of Y	Y $\longrightarrow \phi$	$\tau_y^{-1} y$	τ_y^{-1}

Supporting Table III: OCFFL motif.

Description	Reaction	Propensity function	Rate constant (min^{-1})
Synthesis of S	$\phi \longrightarrow$ S	k_1	k_1
Degradation of S	S $\longrightarrow \phi$	$\tau_s^{-1} s$	τ_s^{-1}
S mediated synthesis of X	S $\longrightarrow$ S+X	$k_2 s$	k_2
Degradation of X	X $\longrightarrow \phi$	$\tau_x^{-1} x$	τ_x^{-1}
X mediated synthesis of Y	X $\longrightarrow$ X+Y	$k_3 x$	k_3
S mediated synthesis of Y	S $\longrightarrow$ S+Y	$k_3' s$	k_3'
Degradation of Y	Y $\longrightarrow \phi$	$\tau_y^{-1} y$	τ_y^{-1}

Supporting Table IV: ACFFL motif.

Description	Reaction	Propensity function	Rate constant (min^{-1})
Synthesis of S	$\phi \longrightarrow$ S	k_1	k_1
Degradation of S	S $\longrightarrow \phi$	$\tau_s^{-1} s$	τ_s^{-1}
S mediated synthesis of X	S $\longrightarrow$ S+X	$k_2 s$	k_2
Degradation of X	X $\longrightarrow \phi$	$\tau_x^{-1} x$	τ_x^{-1}
S & X mediated synthesis of Y	S+X $\longrightarrow$ S+X+Y	$k_3 s x$	k_3
Degradation of Y	Y $\longrightarrow \phi$	$\tau_y^{-1} y$	τ_y^{-1}

Supporting Table V: ICFFL motif.

Description	Reaction	Propensity function	Rate constant (min^{-1})
Synthesis of S	$\phi \longrightarrow$ S	k_1	k_1
Degradation of S	S $\longrightarrow \phi$	$\tau_s^{-1} s$	τ_s^{-1}
S mediated synthesis of X	S $\longrightarrow$ S+X	$k_2 s$	k_2
Degradation of X	X $\longrightarrow \phi$	$\tau_x^{-1} x$	τ_x^{-1}
S & X mediated synthesis of Y	S $\xrightarrow{g(x)^a}$ S+Y	$k_3 g(x) s$	k_3
Degradation of Y	Y $\longrightarrow \phi$	$\tau_y^{-1} y$	τ_y^{-1}

[a] $g(x) = K/(K + x)$, where $K = 1$

[1] Ozbudak EM, Thattai M, Kurtser I, Grossman AD, van Oudenaarden A (2002) Regulation of noise in the expression of a single gene. Nat Genet 31: 69-73.
